# Nursing Perspectives on Palliative and Supportive Care in Paediatric Oncology: A Multicentre Qualitative Study

**DOI:** 10.1002/nop2.70931

**Published:** 2026-10-15

**Authors:** D. Harshitha, Ashmitha Prasad, Krithika S. Rao, Vani Verma, Aishwarya Salian, Arun Ghoshal, Archana Iyengar, Arige Pravalika, Aastha Mishra, Aditi Sakpal, K. Vasudeva Bhat, Véronique Dinand, Gayatri Palat, Naveen Salins

**Affiliations:** ^1^ Department of Palliative Medicine and Supportive Care, Kasturba Medical College Manipal Academy of Higher Education Manipal India; ^2^ Department of Pain and Palliative Medicine MNJ Institute of Oncology and RCC Hyderabad India; ^3^ Palliative and Supportive Care Unit Bai Jerbai Wadia Hospital for Children Mumbai India; ^4^ Department of Pediatric Oncology, Kasturba Medical College Manipal Academy of Higher Education Manipal India

**Keywords:** India, nursing, Paediatric oncology, palliative care, qualitative research, supportive care

## Abstract

**Aim:**

To explore paediatric oncology nurses' perspectives on how supportive and palliative care are understood, delivered, and integrated within routine clinical practice in India.

**Design:**

A multicentre qualitative study informed by a constructivist‐interpretivist paradigm.

**Methods:**

Twenty‐four paediatric oncology nurses from three tertiary cancer centres in India were purposively recruited. Semi‐structured interviews were conducted during 2023–2024, audio‐recorded, transcribed verbatim, translated where necessary, and analysed using reflexive thematic analysis. Analysis was primarily inductive while sensitised by predefined working definitions of supportive and palliative care.

**Results:**

Four interrelated themes were constructed: Clinical Stabilisation and Treatment Continuity; Relational and Communication Work; Embedded Palliative Care; and Emotional Labour. Nurses described continuous surveillance, symptom‐responsive care, communication, navigation, comfort‐focused support, end‐of‐life care, and the emotional work required under resource constraints. ‘Embedded palliative care’ is the researchers' interpretation of practices oriented towards relieving suffering and preserving dignity; participants did not consistently label these activities as palliative care.

**Conclusion:**

Nurses described clinical, relational, supportive, and explicitly palliative practices that responded to suffering across the illness trajectory. The findings indicate areas for service development but do not establish effects on patient safety, treatment continuity, distress, or service outcomes.

**Implications for the Profession and/or Patient Care:**

Practice considerations include strengthening generalist palliative‐care competence, systematic assessment of symptoms and suffering, family communication and navigation, emotional support for nurses, and timely access to specialist palliative care. These approaches require implementation and prospective evaluation.

**Impact:**

The study clarifies how paediatric oncology nurses in three Indian tertiary centres understand and undertake supportive and palliative work, including activities that researchers interpreted as palliative‐oriented even when participants did not use that label.

**Reporting Method:**

The study is reported in accordance with the Standards for Reporting Qualitative Research (SRQR).

**No Patient or Public Contribution:**

No patients, caregivers, or members of the public contributed to the study design, conduct, analysis, interpretation, or manuscript preparation. Nurses participated as research participants.

## 1 Introduction

Cancer is not only a life‐threatening diagnosis but also a prolonged and demanding experience for patients and their families (Rezaei et al. 2024; Woźniak and Iżycki 2014). Beyond the physical effects of the disease and its treatment, cancer often brings emotional distress, uncertainty, social disruption, and financial strain (Rezaei et al. 2024; Li et al. 2024). Even as advances in oncology have improved survival, many patients continue to live with significant symptom burden and psychosocial challenges throughout the course of illness, underscoring the importance of supportive and palliative care alongside curative treatment (Zhi and Smith 2015).

For children with cancer, these challenges are especially profound (Marusak et al. 2018). Prolonged hospitalisations, painful procedures, and disruptions to normal childhood activities affect not only the child but the entire family (Rørbech et al. 2024). Parents are required to make complex decisions while coping with fear, exhaustion, and uncertainty about their child's future (Wu et al. 2025; Sagadiyeva 2021). In the Indian context, families often face additional pressures related to travel, financial constraints, limited social support, and strong cultural expectations to pursue a cure at all costs, even when the child's suffering is considerable (Sagadiyeva 2021; Devaraj et al. 2026).

Nurses are at the heart of the care experience of children with cancer and their families throughout the treatment journey (MacKay et al. 2025; Newman et al. 2020). Paediatric oncology nurses spend extended periods of time with children and their families, often forming close therapeutic relationships through repeated admissions and long treatment courses (Newman et al. 2020; Barratt et al. 2024). They are involved in administering chemotherapy, managing symptoms, comforting children during procedures, and responding to parents' questions and distress, often in moments when families feel most overwhelmed (Weinstein and Henrich 2013). Through this continuous presence, nurses are among the first to recognise subtle changes in a child's condition, emotional needs, or caregiver fatigue (Barratt et al. 2024; Ghaljeh et al. 2024).

At the same time, caring for children with cancer places substantial demands on nurses. They balance technical responsibilities with emotional support while managing heavy workloads and time pressures (Zarenti et al. 2021; De la Fuente‐Solana et al. 2020). Nurses may witness suffering, treatment failure, and death, experiences associated with emotional exhaustion and moral distress (De la Fuente‐Solana et al. 2020; Ventovaara et al. 2021). Communication about symptoms, treatment limitations, or palliative care can also be difficult when families are unprepared or when nurses are uncertain about their role (Devaraj et al. 2026; Newman et al. 2020; Ventovaara et al. 2021; Ehrlich et al. 2020; Mohamed et al. 2022; Sarıkahya et al. 2023; Chuah et al. 2017).

In this study, supportive care refers to preventing and managing the physical, psychological, social, and practical effects of cancer and its treatment across the illness trajectory. Palliative care refers to an active, holistic, needs‐based approach to preventing and relieving serious health‐related suffering and improving quality of life alongside disease‐directed treatment; it may also encompass goals of care and end‐of‐life support (Mohamed et al. 2022; Salins et al. 2024, 2022; Braun and Clarke 2022). These overlapping working definitions sensitised the interview guide and later interpretation. They did not operate as a deductive coding framework, and initial coding remained open to meanings expressed by participants.

Existing qualitative studies describe paediatric oncology nurses' symptom management, communication, and emotional work, but less is known about how these activities interact across Indian tertiary cancer centres with different organisational structures. It is also unclear when nurses themselves understand an activity as palliative care and when researchers interpret supportive or holistic nursing practices as palliative in orientation. This study, therefore, explored nurses' perspectives on how supportive and palliative care are understood, delivered, and integrated in everyday practice, while explicitly distinguishing participants' labels from the researchers' interpretation.

## 2 Methodology

### 2.1 Aim and Study Setting

This study aimed to explore how nurses in paediatric oncology settings in India perceive, deliver, and integrate palliative and supportive care within routine clinical practice. It was conducted across tertiary cancer centres in Manipal, Mumbai, and Hyderabad, representing both private and public institutions, to capture variation in institutional culture, nursing roles, resource availability, and the integration of palliative care across diverse contexts.

This qualitative study was conducted within the same three‐centre research programme as a companion prospective observational study that assessed palliative‐care needs among 150 children with cancer and their families using the Paediatric Palliative Screening Scale (Verma et al. 2026). The datasets were distinct: the present study involved interviews with nurses, whereas the companion study enrolled children and family caregivers.

The qualitative study and companion observational study formed part of the same multicentre research programme, involved the same three institutional sites, operated under the same overarching institutional ethics approvals, and were supported by the same Indian Council of Medical Research grant. They used distinct eligibility criteria, recruitment procedures, participant groups, data collection methods, and datasets; no nurse interview data were included in the companion observational study.

### 2.2 Qualitative Approach and Research Paradigm

The study was guided by a constructivist‐interpretivist paradigm (Fodouop 2024), recognising that accounts of care are shaped by professional socialisation, institutional contexts, and relationships. Reflexive thematic analysis was selected for its theoretical flexibility and suitability for interpreting experiential accounts (Braun and Clarke 2022, 2024). Semi‐structured, one‐to‐one interviews supported detailed exploration across practice contexts—please see Data S1: Interview topic guide for Nurses.

### 2.3 Researcher Reflexivity

Interviews were conducted by project staff who had no supervisory, managerial, or clinical authority over participants and were not known to them before recruitment. Their positioning as members of a palliative‐care research programme may have sensitised them to suffering, relational care, and palliative‐care integration. Reflexive field notes documented contextual observations, assumptions, emotional responses, and emerging interpretations, which were revisited during theme development.

One interviewer was assigned at each participating site. All three interviewers were psychologists by training, had experience conducting qualitative interviews, and were bilingual in English and the principal vernacular language used at their site. Interviews were conducted in English or the participant's preferred local language. Because the site interviewers were bilingual, no interpreter was required.

The interviewers were employed as project staff at the participating centres. They were not known to participants before recruitment and held no supervisory, managerial, or clinical authority over them. Participation or refusal, therefore, had no bearing on participants' employment, appraisal, or clinical responsibilities.

### 2.4 Participant Recruitment and Sampling

Participants were recruited purposively through institutional and professional networks at the three participating centres. Eligible participants were full‐time nurses currently providing paediatric oncology care with at least 2 years of clinical experience. This criterion was intended to ensure sustained exposure to the clinical, relational, supportive, and palliative dimensions of paediatric oncology practice.

Nurses who were not actively engaged in paediatric oncology care at the time of data collection or who declined to provide informed consent were excluded. Sampling sought information‐rich variation in institutional setting, years of experience, qualification, and professional role. Twenty‐four interviews provided a dataset with sufficient experiential range and depth to address the research question across three centres. Sample adequacy was assessed by the richness, relevance, and diversity of accounts generated during concurrent data collection and analysis, rather than by a claim of code or meaning saturation. All participants reported prior exposure to paediatric palliative care; therefore, the study did not compare nurses with and without such exposure.

### 2.5 Data Collection

After written informed consent was obtained, sociodemographic and professional information were collected. Semi‐structured, one‐to‐one interviews were conducted in person, in English or the participant's preferred local language, lasted 30–60 minutes, and were audio‐recorded with permission. The topic guide explored nurses' understanding of supportive and palliative care, integration into daily practice, barriers and facilitators, symptom management, communication with families, and emotional dimensions of care. The guide was iteratively refined as data collection and analysis progressed. Field notes captured contextual and non‐verbal cues.

Interviews at each participating centre were conducted in a side room within the clinic area. The room provided adequate privacy and allowed interviews to proceed without being overheard. Access was limited during interviews, interruptions were minimised, and only the participant and interviewer were present. No interviews were conducted outside the participating institutions.

### 2.6 Participant Profile

A total of 24 paediatric oncology nurses were interviewed across tertiary cancer centres in Manipal, Mumbai, and Hyderabad. All participants were female and ranged in age from 27 to 53 years. No male nurse participated; consequently, the analysis does not represent male nurses' perspectives. Participants included Staff Nurses, Sisters‐in‐Charge, and Nursing Officers. Total clinical experience ranged from 2 to 32 years, and paediatric oncology experience from 2 to 15 years (Table 1).

**TABLE 1:** Socio‐demographic profile of nurses.

Participant	Gender	Age	Qualification	Experience (Yrs)	PPC exposure Yes/No.	Paediatric oncology experience (Years)
P01	Female	37	GNM	12	Yes	2.5
P02	Female	27	GNM	6	Yes	5
P03	Female	39	GNM	17	Yes	2.5
P04	Female	53	GNM	32	Yes	5
P05	Female	27	GNM	5.5	Yes	5
P06	Female	43	GNM	5	Yes	5
P07	Female	38	GNM	8	Yes	8
P08	Female	33	GNM	4	Yes	4
P09	Female	47	GNM	14	Yes	14
P10	Female	34	GNM	10	Yes	10
P11	Female	37	BSC Nursing	8	Yes	8
P12	Female	52	GNM	9	Yes	9
P13	Female	34	BSC Nursing	8	Yes	8
P14	Female	37	GNM	7	Yes	7
P15	Female	32	GNM	7	Yes	7
P16	Female	35	GNM	9	Yes	9
P17	Female	30	GNM	8	Yes	3
P18	Female	30	GNM	2	Yes	2
P19	Female	28	BSC Nursing	3	Yes	3
P20	Female	38	BSC Nursing	20	Yes	15
P21	Female	34	BSC Nursing	8	Yes	5
P22	Female	39	GNM	2	Yes	2
P23	Female	52	BSC Nursing	18	Yes	2
P24	Female	34	GNM	2	Yes	2

*Note:* All participants reported PPC exposure; this variable did not distinguish between formal specialist training and routine clinical contact and is interpreted with caution.

Abbreviations: GNM, general nursing and midwifery; PPC exposure, prior exposure to paediatric palliative care.

The multicentre sample provided contextual breadth across differing organisational environments, patient volumes, resources, and access to specialist palliative care. The centres were used to broaden the range of experiences rather than as separate comparative cases. Theme development, therefore, focused on patterned meanings across the combined dataset; formal centre‐by‐centre comparisons were not undertaken. All 24 participants reported prior exposure to paediatric palliative care, which should be considered when assessing transferability to less‐exposed nursing workforces.

### 2.7 Data Management

Audio recordings were transcribed verbatim in the original interview language (English, Kannada, Hindi, Marathi, or Telugu). Non‐English transcripts were translated into English before coding, and independent back‐translation was used to examine conceptual equivalence. Discrepancies were resolved against the original transcripts and recordings. Coding used the verified English transcripts. Quotations selected for publication were checked against the original‐language transcript and recording, and culturally specific expressions were retained or clarified during interpretation. Transcripts were anonymised and managed in NVivo 14.

The interviewer at each site prepared the English translation of interviews conducted in the local language, with assistance from bilingual members of the site research team. Independent back‐translation was undertaken by a different bilingual research team member who had not prepared the initial translation. Site research team members were proficient in English and the relevant vernacular language and familiar with the local clinical context. Translation discrepancies were discussed within the site team and resolved against the original transcript and audio recording.

### 2.8 Data Analysis

Analysis followed Braun and Clarke's six phases of reflexive thematic analysis: familiarisation, coding, construction of candidate themes, review and development of themes, definition and naming of themes, and analytic writing (Braun and Clarke 2022, 2024). The orientation was primarily inductive at the coding stage: AP coded for meanings expressed in the transcripts without applying a predetermined codebook. The working definitions of supportive and palliative care served as sensitising concepts during later theme development and interpretation, particularly when examining whether participants explicitly identified activities as palliative care or whether the researchers interpreted them as oriented towards the relief of suffering. The analysis attended to the semantic and latent meanings. Ashmitha Prasad (AP) was the primary analyst; AP and Harshitha contributed to thematic interpretation. Themes were iteratively reviewed against coded extracts and the full dataset. They represent interpretive constructions rather than categories objectively discovered or validated through consensus—please see Data S2: Codes, sub‐themes, and themes chart.

### 2.9 Trustworthiness

Analytic rigour was supported through reflexive journaling, a detailed audit trail of coding and theme‐development decisions, and close grounding of interpretations in participants' accounts. An independent qualitative expert participated in peer debriefing at key stages, providing critical dialogue that challenged assumptions and prompted refinement; this was not coding validation or consensus testing. Thick description of the setting, participant characteristics, and institutional diversity helps readers assess transferability.

### 2.10 Ethical Considerations

Ethical approval was obtained in 2023 from the Kasturba Medical College and Hospital Institutional Ethics Committee (IEC‐198/2023), MNJ Institute of Oncology and Regional Cancer Centre (ECR/227/Inst/AP/2013/RR‐19), and Bai Jerbai Wadia Hospital for Children Institutional Ethics Committee (IEC‐BJWHC/61/2023). Before participation, nurses received written and verbal information about voluntary participation, audio‐recording, confidentiality, anonymised quotations, and their rights to decline questions or withdraw without adverse consequences. All participants provided written informed consent. Interviewers monitored for distress; participants could pause or stop the interview or request referral to institutional support. Confidentiality was maintained by using participant identifiers P01–P24 across all transcripts and reporting outputs. Data were stored on password‐protected institutional devices accessible only to the research team.

All three institutional ethics approvals were granted in 2023.

### 2.11 Reporting

The study is reported in accordance with the Standards for Reporting Qualitative Research (SRQR) (O'Brien et al. 2014). The completed checklist is provided as Data S3.

## 3 Results

Four interrelated themes were constructed: Clinical Stabilisation and Treatment Continuity, Relational and Communication Work, Embedded Palliative Care, and Emotional Labour. The first two themes remained close to nurses' descriptions of clinical and relational practice. ‘Embedded Palliative Care’ was a researcher‐generated interpretation: participants explicitly used palliative‐care language for some practices, particularly specialist involvement, refractory symptoms, deterioration, and end‐of‐life care, but did not consistently label routine comfort, psychosocial, or family‐support activities as palliative care. Theme 4 captured the emotional effort described by participants. These interpretations were developed across the combined dataset; centres were not analysed as separate cases.

### 3.1 Theme 1. Clinical Stabilisation and Treatment Continuity

Nurses described clinical stabilisation as grounded in continuous bedside surveillance throughout each shift. Close monitoring was particularly important for younger children unable to communicate pain or discomfort clearly, requiring nurses to interpret behavioural and physical cues, such as altered crying, irritability, reduced intake, and changes in alertness, in relation to ongoing treatment. Observations were carried over shifts through structured handovers that documented overnight symptoms, medication changes, and treatment responses, with prompt reporting to doctors when concerns were identified.

Treatment continuity was not simply a sequence of technical tasks. Across inpatient, outpatient, and daycare settings, chemotherapy, transfusions, intrathecal treatment, supportive therapies, and monitoring required nurses to anticipate risk and connect otherwise fragmented episodes of care. Rapid responses to fever, vomiting, extravasation risk, or transfusion reactions positioned nurses as the immediate interface between protocol and an unpredictable clinical situation. In compressed daycare and outpatient workflows, coordinating investigations, treatment preparation, administration, and discharge served as continuity work: nurses repeatedly adapted formal pathways to ensure children could remain safely engaged in treatment.

Structural and safety constraints intensified this stabilising role. Chemotherapy precautions, line assessment, test dosing, extravasation surveillance, and infection prevention became forms of compensatory vigilance where sanitation, space, medicines, or staffing were limited. Rather than constituting a heterogeneous list of duties, these practices shared a central meaning: nurses absorbed system uncertainty to make treatment possible and safe. Clinical stabilisation thereby created the conditions for the relational and communicative work described in Theme 2 (Table 2).

**TABLE 2:** Subthemes and supporting verbatim for theme 1.

Subtheme	Supporting verbatims
1.1 Bedside observation and early clinical detection	‘However, when we soothe them, they usually calm down. But we still need to observe them to understand why they are crying’. (P01) ‘The patient is a small child and he cannot speak much, a small child cannot speak, he can only cry, but the mother becomes worried that why does this child cry continuously’. (P18) ‘We ensure to observe the children carefully, as we might not know why they are crying or if they are experiencing pain. After administering the injection, we observe them for 10–15 minutes before letting them go’. (P01) ‘If there are problems like severe fatigue or weakness, we monitor the child closely and immediately inform the doctors’. (P05) ‘If a child is sleeping a lot, we need to monitor them. If they are not eating, we must inform them… We monitor their respiration and heart rate’. (P08) ‘If any patient is having high grade fever all night… child is not eating, I have to see all that. First I am seeing that then my round starts…’ (P20) ‘After that we ask everyone, are you okay, has anyone got fever, is there any pain, has any problem occurred…’ (P22) ‘When we come in the morning, we take over from the night staff… who had fever, who was vomiting, who had pain, who was crying…’ (P23) ‘I used to go from bed to bed and first ask if there is any problem with anyone’. (P20) ‘We observe the children, note their needs, and inform our doctors’. (P01)
1.2 Pain and symptom management under clinical uncertainty	‘Actually, Madam, pain is a common issue with chemotherapy…Young children may not be able to communicate their pain effectively. We observe and try to understand why a child is in pain by looking at their reactions and the medications they are taking’. (P01) ‘Most babies, when chemotherapy is given, feel a burning sensation. Most children feel burning and ask us to remove it…Pain is something that is inevitable in a hospital. We can only minimize it to some extent, through play or medication’. (P04) ‘With chemotherapy, we have to insert lines repeatedly. Sometimes veins are difficult to find, causing more pain…Children experience a lot of pain during this process, which makes them dull and withdrawn’. (P05) ‘Some children resist and refuse to cooperate. We have to approach them patiently, speak to them, and console them’. (P06) ‘Pain starts right from the beginning for some children, and for a few it becomes very severe, especially with high‐dose chemotherapy…Oral mucositis becomes so painful that children cannot eat or drink water. Pain relief becomes necessary’. (P08) ‘We do not see any pain from outside. But inside the patient has a lot of pain…When the palliative team assesses and starts narcotic medicine, even a child who used to cry all the time starts playing well’. (P17) ‘If there is mucositis after chemotherapy, the child neither sleeps, nor eats, nor drinks anything’. (P22)
1.3 Treatment continuity across care settings	‘Besides chemotherapy, sometimes we perform blood transfusions, intrathecal injections, or bone marrow procedures. After intrathecal injections, we observe the child for an hour…Then we start the chemotherapy. We secure the IV line first… We need to be careful about extravasation’. (P01) ‘When we start chemotherapy, we check the IV line to ensure there is a proper backflow… If there's no backflow, we replace the line before proceeding…It's not just about administering the medication; it's also about constant observation, communication with parents, and addressing any adverse reactions immediately’. (P05) ‘We administer chemotherapy, blood transfusions, IV antibiotics, and sometimes immunotherapy or IVIG depending on the patient's needs’. (P06) ‘Blood transfusion means… we first check the vitals… if the child has fever or shivering… we stop the blood and inform the doctor’. (P18) ‘After that, we start for injections… IV fluids, chemotherapy, then IT, then the doctor's rounds… discharge procedure… and then handover’. (P23)
1.4 Comfort, dignity, and daily function during intensive treatment	‘Even when children are on a drip, they take the cycle and move around, play, and spend some time comfortably with us’. (P04) ‘We inquire if they have any issues, if they have eaten, how they are feeling, and if they have slept well…If they have mucositis, we provide education about mouthwash, personal hygiene, and gargling’. (P07) ‘Mostly, we advise children to drink a lot of water and keep their mouths clean. If the problem doesn't subside, we admit them and give supportive medicines’. (P03) ‘My round starts by asking if the child has eaten and if the mother has slept. If not, we let the mother rest for some time’. (P20) ‘Sometimes children want only specific food, so we try different ways to make something they will eat’. (P22) ‘We explain to the child and parents, talk to them, reassure them, and then inform the doctors if changes are needed’. (P24) ‘However, once a child becomes familiar with our staff, they recognize us and become more comfortable’. (P01) ‘Sometimes children get scared seeing our uniforms, so we talk to them lovingly or give them a chocolate’. (P18)
1.5 System and safety constraints in nursing work	‘Then we start the chemotherapy. We secure the IV line first. We ensure the IV line is comfortable and not placed in a thin vein. We secure the IV line and administer chemotherapy. We need to be careful about extravasation’. (P01) ‘When we begin chemotherapy, we wear protective gear such as goggles, caps, masks, sterile gloves, and gowns for biosafety…Before administering any drug, we conduct a test dose and wait for about half an hour to monitor for any immediate reactions’. (P05) ‘Cleanliness first, they have very low immunity powers, it is better if the entire area is clean’. (P12) ‘One of the major issues here is related to hospital management, particularly in terms of sanitation and hygiene’. (P10) ‘If we are not properly hygienic, then the treatment will be meaningless’. (P21) ‘Our ward is quite small, with limited resources. We have only 10 beds…Additionally, we are short‐staffed. Having more staff would make it easier to manage’. (P05)

*Note:* (…) indicates parts omitted from the concise verbatim while preserving the original meaning.

### 3.2 Theme 2. Relational and Communication Work in Care Delivery

Relational and communication work functioned as practical infrastructure for care rather than as an optional interpersonal addition. Through repeated explanation, familiarity, counselling, and navigation, nurses helped children and families make sense of illness, tolerate treatment, and remain connected to complex care pathways. The four subthemes, therefore, reflect a shared organising concept: relationships were used to convert information and services into care that families could understand, trust, and continue (Table 3).

**TABLE 3:** Subthemes and supporting verbatim for theme 2.

Subtheme	Supporting verbatims
2.1 Shared understanding of illness, treatment, and prognosis	‘Parents initially feel frustrated and overwhelmed. Over time, we show them children who have completed treatment and explain recovery, and they begin to share their problems with us’. (P03) ‘In the beginning, they don't know anything about the ward or the treatment, but step‐by‐step explanations help them understand better…Some families feel relieved when palliative care is explained, while others struggle to understand it’. (P05) ‘Many parents initially think symptoms like headache or fever are normal and don't recognise them as illness’. (P08) ‘Daily counselling becomes necessary, and families often compare themselves to others in the ward and expect similar outcomes’. (P09) ‘Older children often think that being referred to palliative care means treatment is over’. (P19) ‘Parents may question why palliative care is needed, but once the benefits are explained, they usually agree’. (P10) ‘When parents are told their child may not recover, it helps them prepare emotionally’. (P14) ‘After relapse, parents panic and ask, “I did everything, still the disease came back. What should we do now?”’ (P22)
2.2 Trust, familiarity, and continuity in care relationships	‘Once a child becomes familiar with our staff, they recognise us and become more comfortable’. (P01) ‘Initially they are very nervous and cry a lot, but after supportive communication, they come for the next chemotherapy cycle willingly’. (P02) ‘When children return after earlier admissions, they recognise us and know we are trying to help them’. (P03) ‘With repeated interactions, children and families begin to feel like part of our own family’. (P05) ‘When children see familiar faces, they cooperate better, making rapport essential’. (P01) ‘They remember us and ask about us, and as closeness develops, their fear reduces’. (P02) ‘Even if children cry during a procedure, they quickly return to talk or play with us’. (P03) ‘If we are absent even for a day, children notice and ask where we were’. (P20) ‘Parents feel able to share their inner feelings because we are present with them every day’. (P18) ‘During long admissions, relationships become familial, and we reassure mothers that we will watch over the child’. (P24) ‘Some children return after discharge and still come to talk to us’. (P02) ‘Even years later, former patients and their parents remember us and come to meet us’. (P24) ‘We talk to adolescents like friends, and they share about college, friends, and personal matters’. (P18) ‘Children often share more with nurses than doctors because we remain closer to them’. (P22)
2.3 Counselling and care navigation for treatment continuity	‘Families think one chemotherapy is enough and stop coming. When they don't return, the disease comes back’. (P09) ‘Some children go on maintenance, but after going home they don't take medicines properly and then relapse…At the start, families should be counselled about the disease, treatment, prognosis, and finances before admission’. (P12) ‘At the beginning, patients really need someone to guide them. Otherwise, they leave without proper guidance’. (P17) ‘New patients are confused and overwhelmed, and even when we explain, they may misunderstand’. (P14) ‘Daily counselling is essential because families come with different issues every day…There is a big gap between treatments when families delay visits, and that delay leads to complications’. (P09) ‘Earlier, counsellors used to call families before reviews. That follow‐up system made a big difference, but now it is missing’. (P14) ‘When parents talk to other parents who are ahead in treatment, they are less likely to drop out…We explain hygiene, medicines, when to call the doctor, and not to ignore fever or loose motions’. (P17) ‘We conduct joint sessions where counsellors, dieticians, doctors, and nurses explain everything together, and older patients help demonstrate’. (P20)
2.4 Preferences, expectations, and perceived quality of care	‘Parents have a very positive opinion of palliative care, especially for pain management. When they return, they ask for the same medicine because they don't want their child to suffer’. (P02) ‘When pain is not controlled, palliative care infusions provide major relief’. (P01) ‘When palliative care is explained clearly, families accept it and there is no resistance to pain medicines…Even with IV lines, children play, move around, and sit comfortably with us’. (P04) ‘Families without money can continue treatment here because schemes and trusts support medicines, food, and other needs…After a few days, children start telling us what pain they have and which medicines they are getting’. (P11) ‘Although treatment is free, some families don't return for maintenance because they feel free care is less important’. (P12) ‘Reducing pain and constipation improves how families feel about the care provided’. (P15)

*Note:* (…) indicates material omitted from concise quotations without changing the intended meaning.

Nurses described families arriving with a limited understanding of cancer, often expecting a rapid recovery, with literacy, language, rural background, and stigma further complicating comprehension. In response, nurses undertook ongoing explanatory work, translating diagnoses, treatment rationale, side effects, and medication changes into simple terms, repeated across admissions and adapted to the child's age. Counselling extended beyond the initial diagnosis to include relapse, ICU transfers, and palliative care discussions, with acceptance improving once benefits were clearly explained.

Children were frequently fearful and resistant during early admissions, but with repeated visits, they began to recognise staff and gradually cooperate with procedures. Nurses described building trust through consistent presence, calm communication, and a reassuring manner. Younger children attached quickly, while adolescents were more likely to withdraw, particularly when physical changes or information‐seeking increased distress. Continuity of staff was described as central to reducing fear across repeated and invasive treatments.

Nurses described counselling and navigation as essential because families were often unfamiliar with the long‐term nature of cancer treatment, resulting in missed follow‐ups, premature discharge, and poor maintenance adherence. Navigation support included guidance on treatment schedules, financial aid, accommodation, transport, and coordination with social workers and the palliative care team. Families' perceptions of care quality were shaped primarily by pain relief and affordability, with free services enabling treatment continuation for rural and financially constrained families. This relational continuity, in turn, supported the informal embedding of palliative care practices within routine nursing work.

### 3.3 Theme 3. Embedded Palliative Care

Theme 3 examined the relationship between explicitly identified palliative‐care activities and routine practices that participants described but did not consistently label as palliative care. Explicit accounts included specialist referral, analgesia for refractory symptoms, support during deterioration, and end‐of‐life care. Comfort‐oriented and psychosocial practices were classified by the researchers as palliative in orientation only when the account emphasised relief of suffering, dignity, or quality of life; this interpretation does not imply that all supportive or holistic nursing care is palliative care.

Participants described efforts to preserve children's comfort, dignity, and normalcy during prolonged hospitalisation, including oral care, positioning, nutritional support, dietary modification, movement, and play. They rarely labelled these activities as palliative care. The research team interpreted selected accounts as palliative in orientation when they were directed towards relieving suffering or preserving dignity alongside treatment, while recognising their overlap with supportive and holistic nursing care.

Nurses described end‐of‐life support as part of routine care, staying present with families, offering psychosocial support during deterioration and imminent death, and continuing support after death through family counselling and body handover. Much of this work occurred without specialist input, particularly in the evenings, at night, and on weekends, making it largely unseen yet central to everyday care.

Participants perceived that palliative needs were often recognised when symptoms worsened, or pain became refractory, and described palliative care as commonly associated with the end of treatment. Their accounts suggested a tendency towards reactive rather than anticipatory referral. They also perceived that specialist involvement expanded access to advanced symptom management, multidisciplinary coordination, and community support. These accounts describe perceived processes and do not demonstrate comparative effects of referral (Table 4).

**TABLE 4:** Subthemes and supporting verbatim for theme 3.

Subtheme	Supporting verbatims
3.1 Everyday palliative support within nursing care	‘In end‐of‐life situations, we provide emotional support and try to comfort the families, giving them courage’. (P02) ‘We try to console them, explaining that nothing else could have been done and that their child is no longer suffering’. (P03) ‘Sometimes, a mother might come alone with her child, and suddenly, something happens…Even for us, managing such situations in our busy schedules can be very hard’. (P05) ‘In paediatrics, during the end stage, we need to be attentive to their requests’. (P07) ‘Palliative care means providing psychological support to patients and ensuring they experience minimal pain’. (P16) ‘Apart from pain management, the patient should be happy till the last moment’. (P17) ‘After death, till the body is handed over, I go with them’. (P20)
3.2 Late recognition of palliative needs	‘Many people come to us only after their loved ones are in the final stages. If they had been informed earlier, they might have had better control over the situation’. (P13) ‘They think, ‘Why do I need this? I don't even have pain’. What they don't understand is that pain can come at any time…They don't realize that palliative care is also involved and provides crucial support for the patient’. (P09) ‘Even though there is a perception that palliative care is only for end‐of‐life situations, it is not always the case’. (P01) ‘If there's a treatment failure, they may not accept it… they want to continue treatment rather than switch to palliative care’. (P07) ‘Older children think being sent to palliative care means treatment is over’. (P19) ‘Why do I need palliative care, it was fine right now. His reports were fine right now…Let the senior doctors come first… after consultation with all the doctors, they become ready’. (P24)
3.3 Care reconfiguration following palliative involvement	‘There are many NGOs in our department who help families, and because of them even uneducated patients complete the 2–2½‐year treatment well and go home’. (P17) ‘The palliative team even goes to patients' homes, arranging things like oxygen or an ambulance if needed, and continues supporting them as long as the patient is alive’. (P19) ‘Social workers help families get cheaper medicines through ClearPoint or Tata funds, guiding them about documents like ration card and Aadhaar so they don't waste money and time buying at full cost…For outstation families, social workers arrange free, safe rooms and a vehicle so parents can stay nearby and come any time there is a problem’. (P20) ‘A paediatric day‐care unit was opened two years ago so children can get outpatient chemotherapy and reduce admission costs’. (P01) ‘In my opinion, almost everything is provided here nutrition, pain management, and even financial help through social workers which is very helpful for families’. (P06) ‘Poor patients from villages get free treatment through Arogyashree and different trusts that provide money, food, and clothes, so they can continue cancer care’. (P11)

*Note:* (…) indicates parts omitted from the concise verbatim while preserving the original meaning.

### 3.4 Theme 4. Emotional Labour in Nursing Practice

Nurses described emotional labour as sustained, regulated work required to function within emotionally intense paediatric oncology settings, shaped by repeated exposure to suffering, loss, and system pressures. Emotional regulation, cumulative strain, coping, and structural constraints were described as inseparable from routine clinical practice. The subthemes under theme 4 and their supporting verbatim are presented in Table 5.

**TABLE 5:** Subthemes and supporting verbatim for theme 4.

Subtheme	Supporting verbatims
4.1 Emotional labour in everyday clinical care	‘The child is in front of you, you cannot cry in front of him, because he will be more scared’. (P20) ‘It feels difficult seeing children in such conditions. I often think about how it would be if my own children faced these problems. We also form a bond with the children. We need to listen to the pain of their families too’. (P02) ‘Seeing children of the same age as our own children is genuinely difficult. Detaching emotionally is not possible’. (P04) ‘It's very hard when something bad happens. We feel deeply sad, but we can't show it to the parents. We explain everything with care and tell them to stay strong’. (P13) ‘Even if we feel emotional inside, we still have to continue our duty’. (P16)
4.2 Cumulative emotional strain, moral distress, and coping	‘When a death occurs in our ward… it affects us so much that we lose our appetite and sleep, and everything is affected…We have to accept the situation. There is no other way. That is how we manage’. (P07) ‘We used to suffer a lot in the beginning, sometimes for a week. Now with experience, we move past it within a day or two. It has become a habit’. (P15) ‘In the beginning, it was very disturbing. Seeing these innocent children suffering was overwhelming… later, as the repetition kept happening, the fear reduced a bit’. (P17) ‘Initially, I was shocked seeing so many children with cancer. Now I have become accustomed to it, but that first phase was very difficult’. (P12) ‘Despite giving patients all the care and treatment possible, we still aren't able to save them. That is heartbreaking’. (P02) ‘Initially, I used to cry along with them. Later I understood this is my job and I have to carry on’. (P10) ‘When someone crashes after long treatment, it feels like we are losing somebody… that day we don't think positively’. (P20) ‘Even after going home, I used to think about why this happens only to children’. (P18)
4.3 System and staffing pressures shaping emotional labour	‘For 100 patients, there are only 5–6 nursing staff. It's impossible to provide the level of care we should’. (P12) ‘My interaction with a child may be only 2–3 minutes during cannulation. In that time, we can't have a full conversation’. (P09) ‘Sometimes we need to act very quickly, like when a child develops a fever. We must start antibiotics within half an hour’. (P06) ‘Once the diagnosis happens, families who are not from Bombay have no arrangement for living or food… we arrange food and guide them for bathing and accommodation’. (P20) ‘Gents are not allowed inside. They don't bathe for two to three days… we have to guide them to paid public washrooms’. (P17) ‘Sometimes we allow extra attendants because of language problems or young mothers… we have to decide who needs more’. (P23) ‘Once a mother had nothing to eat at night. I shared my tiffin. She said she had no money and was new to Mumbai’. (P22) ‘Dieticians advise special diets, but there is no free supply. We manage with what we have’. (P08) ‘Accommodation, hygiene, resting space, these are basic needs. Without this, everything becomes harder’. (P24)

*Note:* (…) indicates parts omitted from the concise verbatim while preserving the original meaning.

Nurses described consciously suppressing visible distress during demanding clinical interactions to remain present, composed, and functional. Close relationships with children and families, sometimes intensified by nurses' own parental identifications, made emotional neutrality difficult. Managing parental uncertainty, stigma, and repeated questioning required controlled and measured responses throughout each shift.

Early experiences were marked by shock, sadness, and moral discomfort, particularly when caring for children with whom strong bonds had formed. Sustained exposure accumulated over time, contributing to emotional exhaustion, sleep disturbance, and periods of demotivation. Nurses described adapting not through detachment but by normalising uncertainty and accepting limits to control, drawing meaning from recoveries, survivor follow‐ups, and parental gratitude to sustain continued engagement.

System pressures extended emotional labour beyond bedside care into managing families' basic survival needs, food, accommodation, transport, and hygiene for outstation caregivers arriving without arrangements. Staffing shortages and high patient loads further constrained the space available for emotional support. Nurses described system failures as not simply limiting emotional care but actively generating additional emotional tasks, embedding emotional labour within the structural work required to keep families able to remain in treatment. Taken together, these four dimensions of nursing work collectively maintained continuity of care within an under‐resourced system.

Across the four themes, nurses' accounts linked clinical surveillance, relational work, supportive and palliative practices, and emotional labour. The research team interpreted these dimensions as an informal system‐of‐care stabilisation. This conceptual model describes patterned meanings in the interviews; it does not establish causal effects on treatment continuity, safety, distress, or service outcomes.

## 4 Discussion

Nurses' accounts portrayed bedside surveillance, early detection, and cross‐setting coordination as an informal safety function within resource‐constrained paediatric oncology care, consistent with literature on nursing surveillance and paediatric oncology nursing in low‐ and middle‐income countries (Kim and Cho 2023; Radhakrishnan et al. 2022). Participants perceived these practices as important for responding to clinical change and maintaining continuity across inpatient, outpatient, and daycare settings. The study did not measure patient safety events, treatment delays, or abandonment; any influence on these outcomes should therefore be treated as a plausible implication that warrants evaluation.

Participants described nurses as relational bridges who translated medical information and helped families navigate treatment, finances, accommodation, transport, and documentation (Cheng et al. 2019; Bluebond‐Langner et al. 2007). They perceived that familiarity and calm communication reduced fear and supported cooperation, but procedural distress and cooperation were not measured outcomes (Rennick and Rashotte 2009). Navigation may help families remain connected to treatment, particularly where abandonment is a recognised concern, but this study cannot establish such an effect (Arora et al. 2007). Families were described as valuing visible comfort and relief of suffering, consistent with literature on symptom burden at the end of life (Wolfe et al. 2000).

The relational findings can also be interpreted through a trauma‐informed lens. Repeated procedures, uncertainty, loss of control, altered appearance, and disrupted routines may threaten psychological safety. Participants described consistent presence, age‐appropriate explanation, reassurance, and preservation of normalcy; these practices are conceptually aligned with trauma‐informed paediatric oncology nursing (Çınar Özbay et al. 2025). This is a researcher‐generated interpretation: participants did not report using a formal trauma‐informed model, and the study did not evaluate its effects.

A central interpretation is that palliative‐oriented support may coexist with disease‐directed treatment in everyday oncology nursing. Participants explicitly described palliative care in relation to refractory symptoms, specialist involvement, deterioration, and end‐of‐life care. The researchers additionally interpreted selected comfort, dignity, and family‐support practices as palliative in orientation, while recognising their overlap with supportive and holistic nursing care (Salins et al. 2022; Hasson et al. 2020). Participants perceived formal referral as late and associated this with beliefs that palliative care signalled loss of hope; this pattern is consistent with literature on early integration and referral (Mohamed et al. 2022; Cheng et al. 2019; Hasson et al. 2020). Specialist services were described as providing additional symptom management and multidisciplinary resources, but the present study did not measure their effects. Contemporary frameworks support needs‐based palliative care alongside oncology treatment (World Health Organization 2020; Knaul et al. 2018).

Emotional labour was described as a routine requirement of paediatric oncology nursing. Participants reported maintaining composure while encountering suffering, deterioration, and death, often without formal psychological support. They linked moral tension to staffing and resource constraints, consistent with the literature on ethical climate, burnout, and palliative care work (Zarenti et al. 2021; De la Fuente‐Solana et al. 2020; Ventovaara et al. 2021; Sarıkahya et al. 2023). These accounts indicate potential workforce risks, but exhaustion, attrition, patient safety, and service sustainability were not measured and should not be presented as demonstrated outcomes.

The findings support several considerations for practice and implementation research. Services could consider strengthening generalist palliative‐care and trauma‐informed competencies, referral pathways, reflective supervision, and navigation support. These interventions were not shown in this study to improve outcomes; their acceptability, feasibility, fidelity, resource requirements, and effects should be evaluated prospectively across different service contexts.

### 4.1 Implications for Nursing Practice

Paediatric oncology services may consider defining generalist palliative‐care competencies for nurses, including developmentally appropriate assessment of symptoms and suffering, communication with children and families, and recognition of indications for specialist referral. Continuity and navigation roles should be clarified within multidisciplinary teams, while structured opportunities for reflective supervision and psychological support may help address the emotional demands described by participants. Trauma‐informed principles may offer a useful framework for communication and procedural care. Each consideration requires local adaptation, formal implementation, and outcome evaluation; the present qualitative findings do not demonstrate effectiveness.

### 4.2 Strength and Limitations

Data from three tertiary cancer centres provided contextual breadth across private and government institutions. Purposive recruitment yielded variation in age, experience, qualifications, roles, and institutional contexts, while semi‐structured interviews elicited detailed accounts of experience. Reflexive journaling, peer debriefing, an audit trail, and SRQR‐informed reporting supported analytic transparency. Reflexive thematic analysis enabled interpretation of both explicit accounts and underlying meanings without treating coding agreement as a test of validity.

Several limitations should guide interpretation. Recruitment through institutional and professional networks may have favoured nurses with a positive orientation towards palliative care. All participants were women, and all reported prior exposure to paediatric palliative care; perspectives of male nurses and nurses without such exposure are therefore absent. The predominance of private‐sector participants and restrictions on tertiary centres limit transferability to government, district, community, and non‐specialist services. The multicentre design increased contextual breadth, but centres were not analysed as separate cases, and formal centre‐level comparisons cannot be made. Coding of translated English transcripts may have reduced linguistic nuance despite bilingual verification and back‐translation. Findings are not statistically generalisable; readers should assess transferability based on contextual similarity.

## 5 Conclusion

This study describes how paediatric oncology nurses understood and enacted clinical surveillance, communication, care navigation, comfort‐focused support, explicit palliative care, and emotional labour across three Indian tertiary centres. The findings distinguish participants' own use of palliative‐care language from the researchers' interpretation of selected routine practices as palliative in orientation. The findings suggest opportunities to strengthen generalist palliative‐care competencies, symptom and suffering assessment, family communication, support for nurses, and timely specialist referral. These are practice considerations that require implementation and prospective evaluation rather than effects demonstrated by this study.

## Author Contributions

**D. Harshitha:** investigation, writing – original draft, methodology, validation, writing – review and editing, formal analysis, software, project administration, data curation. **Ashmitha Prasad:** writing – original draft, investigation, methodology, validation, writing – review and editing, software, formal analysis, project administration, data curation. **Krithika S. Rao:** conceptualization, investigation, methodology, writing – review and editing, project administration, supervision, resources. **Vani Verma:** investigation, writing – review and editing, methodology, validation, software, formal analysis, project administration, data curation, resources. **Aishwarya Salian:** investigation, methodology, validation, writing – review and editing, project administration, resources, data curation. **Arun Ghoshal:** writing – original draft, methodology, writing – review and editing, project administration, data curation, supervision, resources. **Archana Iyengar:** conceptualization, methodology, writing – review and editing, software, project administration, supervision, resources. **Arige Pravalika:** investigation, methodology, validation, writing – review and editing, formal analysis, project administration, data curation. **Aastha Mishra:** investigation, methodology, validation, writing – review and editing, formal analysis, project administration, data curation. **Aditi Sakpal:** investigation, writing – review and editing, validation, methodology, formal analysis, project administration, data curation. **K. Vasudeva Bhat:** conceptualization, writing – review and editing, methodology, software, project administration, supervision, resources. **Véronique Dinand:** conceptualization, methodology, writing – review and editing, software, project administration, supervision, resources. **Gayatri Palat:** conceptualization, methodology, writing – review and editing, software, project administration, supervision, resources. **Naveen Salins:** conceptualization, funding acquisition, methodology, writing – review and editing, software, project administration, supervision, resources.

## Funding

This study was supported by the Indian Council of Medical Research, New Delhi (Grant No. 5/13/18/NS/ICRC/2022/NCD‐III; E‐Office ID 135492), awarded to Dr. Naveen Salins as Principal Investigator. The funder had no role in participant recruitment, data collection, analysis, interpretation, manuscript preparation, or the decision to submit the manuscript for publication.

## Ethics Statement

Ethical approval was obtained from all three participating sites: Kasturba Medical College & Hospital IEC: IEC‐198/2023. MNJ Institute of Oncology & RCC: ECR/227/Inst/AP/2013/RR‐19. Bai Jerbai Wadia Hospital for Children IEC: IEC‐BJWHC/61/2023.

## Consent

All participants provided written informed consent prior to participation. Confidentiality was maintained through participant identifiers (P01–P24) in all transcripts and reporting outputs. Data were stored securely on password‐protected institutional devices with access restricted to the research team.

## Conflicts of Interest

The authors declare no conflicts of interest.

## Supporting information


**Data S1:** Interview topic guide for Nurses.

**Data S2:** Codes, sub‐themes, and themes chart.

**Data S3:** Standards for reporting qualitative research (SRQR) checklist.

## Data Availability

The interview data are not publicly available because they contain potentially identifiable contextual information. Reasonable requests for access to appropriately de‐identified data may be considered by the corresponding author, subject to institutional ethics approval and applicable consent and data‐governance requirements.
