# Supplementary material for: Nursing Perspectives on Palliative and Supportive Care in Paediatric Oncology: A Multicentre Qualitative Study
Source: Nurs Open. 2026 Oct 15;13(10):e70931. doi: 10.1002/nop2.70931 (PMC13673012; doi:10.1002/nop2.70931)
Supplement: Supplementary file 1 [file NOP2-13-e70931-s001.docx]

**Supplementary material I: Interview topic guide for Nurses**

| SL NO | QUESTIONS | PROBES |
| --- | --- | --- |
| 1 | Can we talk about your experience in caring for children with cancer? | What does your typical day look like?  How many years of experience working with children with cancer?  How do you manage your day in caring for children with cancer? |
| 2 | Can we talk about your experience in caring for children with cancer? | Challenges faced while working with cancer patients and caregivers  Success of the treatment: Personal insights |
| 3 | Could you share your opinion on the needs of the patient and caregiver? | Specific approaches or strategies, such as physical, emotional, and social needs |
| 4 | How do you use palliative care ideas to help the care of children with cancer? | Aware of the palliative care  View about the palliative care  Do you think it's beneficial for the child with cancer? |
| 5 | Can we talk about some of the challenges you face in providing palliative care to children with cancer? | Resistance to the treatment  Challenges faced while providing the treatment  Experience in understanding palliative care |
| 6 | Can we discuss how you approach communication with children with cancer and their families about palliative care options? | How do you initiate the conversation about palliative care with families? |
| 7 | Can we talk about the strategies used to communicate palliative care options to children with cancer and their families? | Any specific strategies  Any difficulties faced during the communication*.*  Any resistance from the family members |
| 8 | Are there any resources or services that you routinely recommend to families for additional support? |  |
