# Supplementary material for: Nursing Perspectives on Palliative and Supportive Care in Paediatric Oncology: A Multicentre Qualitative Study
Source: Nurs Open. 2026 Oct 15;13(10):e70931. doi: 10.1002/nop2.70931 (PMC13673012; doi:10.1002/nop2.70931)
Supplement: Supplementary file 2 [file NOP2-13-e70931-s002.docx]

**Supplementary material III. Standards for Reporting Qualitative Research (SRQR) Checklist**

*Nursing Perspectives on Palliative and Supportive Care in Paediatric Oncology: A Multicentre Qualitative Study*

| Topic | No. | SRQR reporting requirement | Where reported in the manuscript |
| --- | --- | --- | --- |
| Title | 1 | Concise description of the study topic; identifies the qualitative nature or approach. | Title, p. 1: identifies the study as a multicentre qualitative study. |
| Abstract | 2 | Summary of the background/purpose, methods, results, and conclusions, in the journal’s required format. | Abstract, pp. 1–2: Aim, Design, Methods, Results, Conclusion, Implications, Impact, Reporting Method, and No Patient or Public Contribution. |
| Problem formulation | 3 | Description and significance of the problem, supported by relevant theory or empirical literature. | Introduction, pp. 3–4: clinical and family burden, nursing role, care challenges, definitions, evidence gap, and study rationale. |
| Purpose or research question | 4 | Purpose of the study and any specific objectives or questions. | End of Introduction and Aim and Study Setting, p. 4. |
| Qualitative approach and research paradigm | 5 | Qualitative approach and guiding theory or paradigm, with rationale where relevant. | Qualitative Approach and Research Paradigm, p. 5; Data Analysis, pp. 9–10: constructivist–interpretivist paradigm and reflexive thematic analysis. |
| Researcher characteristics and reflexivity | 6 | Researcher attributes, relationships, assumptions, or positioning that may influence the study. | Researcher Reflexivity, pp. 5–6; Data Analysis, p. 10: interviewer backgrounds, employment and prior relationships, authority, positioning, reflexive notes, and analyst roles. |
| Context | 7 | Study setting and contextual factors relevant to the findings. | Aim and Study Setting, pp. 4–5; Researcher Reflexivity and Data Collection, pp. 5–7: three tertiary centres, institutional variation, interviewer/site arrangements, and interview setting. |
| Sampling strategy | 8 | How and why participants or data sources were selected; criteria for stopping data collection. | Participant Recruitment and Sampling, pp. 6–7: purposive recruitment, eligibility/exclusion criteria, variation sought, and information-power/sample-adequacy rationale. |
| Ethical issues pertaining to human subjects | 9 | Ethics approval and consent; confidentiality and data-security procedures; relevant risks and protections. | Ethical Considerations, pp. 10–11: three committee approvals, 2023 approval year, written consent, voluntary participation, recording, confidentiality, withdrawal rights, distress protocol, referral, identifiers, and secure storage. |
| Data collection methods | 10 | Types of data collected and details of collection procedures, including dates, iterative processes, and rationale. | Data Collection, pp. 6–7: in-person semi-structured interviews during 2023–2024, languages, duration, audio-recording, field notes, iterative topic-guide refinement, privacy, and location. |
| Data collection instruments and technologies | 11 | Description of instruments, guides, and recording technology, including their development or modification. | Qualitative Approach, p. 5, and Data Collection, pp. 6–7: semi-structured topic guide (Supplementary Material I), audio-recording, and iterative refinement. |
| Units of study | 12 | Number and relevant characteristics of participants, documents, or events included in the analysis. | Participant Profile and Table 1, pp. 7–9: 24 female paediatric oncology nurses; age, qualification, role, experience, site context, and prior palliative-care exposure. |
| Data processing | 13 | Transcription, translation, data entry and management, security, verification, coding preparation, and anonymisation. | Data Management, pp. 9–10; Ethical Considerations, pp. 10–11: verbatim transcription, translation/back-translation, discrepancy resolution, quotation checks, NVivo 14, anonymisation, identifiers, and password-protected storage. |
| Data analysis | 14 | Process used to develop interpretations or themes; researchers involved; analytic approach and rationale. | Data Analysis, pp. 9–10: Braun and Clarke’s six phases, primarily inductive coding with sensitising concepts, semantic/latent interpretation, AP as primary analyst, and AP and Harshitha in theme development. |
| Techniques to enhance trustworthiness | 15 | Methods used to strengthen credibility or trustworthiness, with rationale where relevant. | Researcher Reflexivity, p. 5; Data Collection, p. 7; Trustworthiness, p. 10: reflexive field notes/journaling, audit trail, grounding in accounts, peer debriefing, and thick description. |
| Synthesis and interpretation | 16 | Main findings, including themes, interpretations, models, or explanatory accounts. | Results, pp. 11–27: four themes and subthemes; integrative interpretation and informal system-of-care stabilisation model, pp. 27–28. |
| Links to empirical data | 17 | Evidence supporting analytic claims, such as quotations, excerpts, field notes, or other data displays. | Results narrative and Tables 2–5, pp. 12–27: extensive participant quotations labelled P01–P24. |
| Integration with prior work, implications, transferability, and contribution | 18 | Relationship of findings to prior literature or theory; implications, applicability, and contribution. | Discussion, pp. 28–29; Implications for Nursing Practice, pp. 29–30; Strengths and Limitations and Conclusion, pp. 30–31. |
| Limitations | 19 | Limitations of the evidence and of the study methods, including implications for interpretation. | Strengths and Limitations, p. 30: recruitment, sample composition, sector/setting, non-comparative design, translation nuance, and transferability. |
| Conflicts of interest | 20 | Potential sources of influence and how they were addressed. | Declarations—Conflict of Interest, p. 31: authors declare no conflicts of interest. |
| Funding | 21 | Sources of funding or other support and the funder’s role in the study. | Declarations—Funding, p. 31: Indian Council of Medical Research grant number and statement that the funder had no role in the research or publication decision. |

**SRQR source:** O’Brien BC, Harris IB, Beckman TJ, Reed DA, Cook DA. Standards for reporting qualitative research: a synthesis of recommendations. Acad Med. 2014;89(9):1245–1251. doi:10.1097/ACM.0000000000000388.
