# Supplementary material for: Nursing Perspectives on Palliative and Supportive Care in Paediatric Oncology: A Multicentre Qualitative Study
Source: Nurs Open. 2026 Oct 15;13(10):e70931. doi: 10.1002/nop2.70931 (PMC13673012; doi:10.1002/nop2.70931)
Supplement: Supplementary file 3 [file NOP2-13-e70931-s003.docx]

| **Supplementary material II: Codes, sub-themes, and themes chart** | | |
| --- | --- | --- |
| **Themes** | **Subthemes** | **Codes** |
| Theme 1. Clinical Stabilisation in Paediatric Oncology Nursing Care | 1.1 Bedside observation and early clinical detection | Difficulty differentiating causes of crying in children Proactive problem-solving and vigilant observation in care Bedside preparedness enhancing patient care Nursing preparedness, comforting presence, and self-reliant task management Nursing role and contribution in clinical readiness Early detection of discomfort and palliative needs by nurses Risk of error in pre-chemotherapy checks Risk of iv-related adverse events Standardization and safety in nursing and chemotherapy procedures |
|  | 1.2 Pain and symptom management under clinical uncertainty | Difficulty managing pediatric pain and procedures Unpredictable and variable treatment side effects Individual differences in side effect experiences Age-related sensitivity to pain in pediatric patients Variability in disease progression and response Physical side effects of chemotherapy in children Incomplete pain relief despite medication use Perception of pain as greater burden than disease Mixed clinical outcomes in pediatric oncology treatment Mixed treatment outcomes observed by nurses Relationship quality as mediator of pain perception  Need for enhanced and specialized pediatric pain management approaches |
|  | 1.3 Treatment continuity across care settings | Nursing role in chemotherapy and supportive procedural care Nursing role in managing chemotherapy and medication side effects Nursing role in pain management during chemotherapy Nursing role in symptom relief and management of chemotherapy complications Nursing-led chemotherapy administration and post-treatment monitoring Extended nursing involvement in specialized procedures Structured educational and procedural nursing practices Observed and anticipated treatment discontinuity risks Cycle delays due to multi-step process Nursing-led pain management as central palliative function |
|  | 1.4 Comfort, dignity, and daily function during intensive treatment | Child distress from painful procedures Effective relief of pain and physical discomfort in children Relief of pain and emotional distress in children Relief of pain and physical discomfort in children Improved comfort and activity level after pain relief Improved dignity and comfort through ongoing nursing care Enhanced child comfort through familiarity and gentle nursing practices Stabilization of child health through acute symptom management Symptom control during and after chemotherapy Symptom relief and stabilization of child health through continuous nursing support |
|  | 1.5 System and safety constraints in nursing work | Infrastructure limitations for palliative procedures in opd Need for infrastructure development to support pediatric day care Medicine supply gaps affecting continuity of symptom relief Need for enhanced infection-control and cleanliness measures Risk management for infections in immunocompromised children Occupational exposure risks in oncology nursing Prevention of treatment-related complications |
| Theme 2. Relational and Communication Work in Care Delivery | 2.1 Shared understanding of illness, treatment, and prognosis | Gaps in health literacy among caregivers Difficulty accepting prognostic information among parents Inadequate explanation prior to referral Delayed acceptance of treatment due to limited health literacy Parental denial and emotional resistance during diagnosis Clear explanation approach by nurses Child-centered and age-appropriate communication strategies Confidence in delivering palliative care communication Emotional preparedness through information Need for early parental counseling on diagnosis and prognosis Structured and transparent communication training and risk discussion for providers Inadequate counselling integration across care pathway  Parental emotional distress from poor prognosis communication |
|  | 2.2 Trust, familiarity, and continuity in care relationships | Building familiarity as a coping mechanism in care Consistent staffing to build trust with children Rapport-building strategies for pediatric care Continued rapport-building after discharge Emotional support bridging between inpatient and family Gentle and supportive nursing care during vulnerable phases Nursing presence and facilitation in pediatric oncology care Parental isolation during non-duty hours Reduced emotional distress and fear among families Reduced procedural fear in children over time Short-term relief of child distress through nurse support Empathic communication and emotional connection with children  Empathic support and communication with families Supportive nursing communication during procedures Reduced emotional distress and fear among families |
|  | 2.3 Counseling and care navigation for treatment continuity | Poor treatment adherence during maintenance phase  structured pre-treatment orientation for caregivers Orientation for caregivers Comprehensive parental education and counseling by nurses Need for structured follow-up and adherence counseling Parental counseling and positive reinforcement to foster treatment acceptance Nursing role in patient navigation Nursing participation in multidisciplinary care and counseling Improved treatment continuity through nursing and family support mechanisms Collaborative and supportive interprofessional practice Improved caregiver understanding and child cooperation through regular counselling Improved family acceptance and trust toward palliative care through communication Improved family adjustment, coping, and understanding through continued support Improved family confidence in managing treatment effects Improved family cooperation and communication through rapport and confidence-building Improved safety and understanding through clear medication guidance Improved safety and understanding through early counselling |
|  | 2.4 Preferences, expectations, and perceived quality of care | Parental preference for proven pain relief Perceived quality and value in free oncology services Dependence on parents for managing child cooperation Child-centered behavioral adjustment Empowerment through knowledge sharing Positive reframing of child adaptation by nurse |
| Theme 3. Palliative Care Practices Within Routine Nursing Work | 3.1 Everyday palliative support within nursing care | Absence of continuous psychosocial support coverage Inadequate psychological support coverage across shifts Continuous nurse-led psychosocial support across shifts Ethical commitment to relieve suffering till the end Nursing and family support continuity until and beyond end-of-life |
|  | 3.2 Late recognition of palliative needs | Delayed symptom management due to limited awareness Limited awareness of palliative care among nurses Misconception of palliative care as end-of-life care Nursing role in identifying and reporting palliative needs Acceptance of palliative care influenced by pain experience and treatment outcomes |
|  | 3.3 Care reconfiguration following palliative involvement | Limited access to integrated pediatric palliative support services Availability of specialized palliative care services Positive integration and attitude toward palliative care Collaborative interprofessional practice between nurses, doctors, and palliative teams Systematic referral pathways from oncology and nursing to palliative care Role of palliative care in symptom and emotional support Access to pain and symptom management through referral Integrated symptom relief through palliative care Integration of diagnostic and palliative services within government hospital Improved accessibility to comprehensive and supportive pediatric oncology services Reduced financial burden through supportive and outpatient care |
| Theme 4. Emotional Labour in Nursing Practice | 4.1 Emotional labour in everyday clinical care | Emotional identification with the child Emotional strain from seeing child distress Pain-related emotional reactions in children Emotional distress in children and families during treatment Emotional resilience while managing parental distress Emotional regulation during clinical encounters  Emotional strain from empathic involvement with pediatric patients Emotional strain from witnessing parental distress Emotional distress from illness awareness in adolescents |
|  | 4.2 Cumulative emotional strain, moral distress, and coping | Emotional and psychological fatigue from continuous pediatric exposure Emotional exhaustion from repeated pediatric death exposure Emotional strain from witnessing child mortality Emotional strain from end-of-life exposure Emotional strain from exposure to pediatric illness Emotional adjustment to pediatric oncology and recurrent mortality Acceptance of clinical reality and treatment uncertainty Emotional desensitization from continuous pediatric exposure Reduced emotional engagement due to workload and fatigue Adjustment to dual roles of work and home Professional adjustment and normalization of emotional strain Emotional and professional adaptation through experience Collaborative teamwork as emotional support for nurses Spiritual coping Psycho-social counseling and stress-relief support for oncology staff Emotional reward through parental gratitude Positive emotional reinforcement through child recovery Therapeutic fulfillment and professional satisfaction in care delivery Professional growth and emotional balance through experience Higher perceived treatment success in pediatric cases Emotional fulfillment and resonance from positive pediatric outcomes |
|  | 4.3 System and staffing pressures shaping emotional labour | High workload and workflow gaps in nursing care delivery Staff shortages limiting consistent and psychosocial care Physical and operational strain among nursing staff Dependence on doctors for care and referral decisions Institutional environment and hierarchical dynamics in oncology care Limited emotional support systems for nurses Need for strengthening nursing workforce capacity Work setting in specialized pediatric units Collaborative task sharing between doctors and nurses Effective problem-solving through team support Physical and operational strain among nursing staff Financial barriers affecting treatment continuity Insufficient caregiver accommodation and hygiene facilities Caregiver nutrition and wellbeing monitoring Comprehensive caregiver welfare and daily living support Creation and nursing contribution in pediatric oncology day care unit Dedicated counselor deployment in pediatric oncology wards Structured day-engagement options for caregivers and children Trained volunteer or respite care services Reduced motivation for adherence in free care settings Unmet emotional support needs among families |

| **Subthemes** | **Verbatims - main** | **Verbatims - supplementary** |
| --- | --- | --- |
| 1.1 Bedside observation and early clinical detection | “However, when we soothe them, they usually calm down. But we still need to observe them to understand why they are crying.” (P01) “Yes, we talk to them kindly and approach them calmly. Sometimes they cry, but after we console them, they usually calm down.” (P06) “The patient is a small child and he cannot speak much, a small child cannot speak, he can only cry, but the mother becomes worried that why does this child cry continuously.” (P18) “We ensure to observe the children carefully, as we might not know why they are crying or if they are experiencing pain. After administering the injection, we observe them for 10–15 minutes before letting them go.” (P01) “If there are problems like severe fatigue or weakness, we monitor the child closely and immediately inform the doctors.” (P05) “If a child is sleeping a lot, we need to monitor them. If they are not eating, we must inform them… We monitor their respiration and heart rate.” (P08) “If any patient is having high grade fever all night… child is not eating, I have to see all that. First I am seeing that then my round starts…” (P20) “After that we ask everyone, are you okay, has anyone got fever, is there any pain, has any problem occurred…” (P22) “When we come in the morning, we take over from the night staff… who had fever, who was vomiting, who had pain, who was crying…” (P23) “I used to go from bed to bed and first ask if there is any problem with anyone.” (P20) “We observe the children, note their needs, and inform our doctors.” (P01) | “As soon as we come, we segregate everything cannulas, medication, what treatment they need, what has been stopped, all of it.” (P09) “When children come, the first thing they do is undergo blood tests. We take their samples, label them, and send them for reporting.” (P03) “They undergo blood investigations here. When we arrive, first, we make the beds and attend to the children.” (P01) “Then my round starts asking them if they had breakfast, is mother sleeping.” (P20) “The over takes half an hour.” (P23) |
| 1.2 Pain and symptom management under clinical uncertainty | “Actually, Madam, pain is a common issue with chemotherapy. When starting certain medications like Dexona, throat pain may occur, and some children may have uncontrolled pain.” (P01) “Young children may not be able to communicate their pain effectively. We observe and try to understand why a child is in pain by looking at their reactions and the medications they are taking.” (P01) “Working with children was initially intimidating. Young children don’t understand instructions, and they cry or resist when we try to give injections or take blood samples.” (P03) “Most babies, when chemotherapy is given, feel a burning sensation. Most children feel burning and ask us to remove it.” (P04) “Pain is something that is inevitable in a hospital. We can only minimize it to some extent, through play or medication.” (P04) “With chemotherapy, we have to insert lines repeatedly. Sometimes veins are difficult to find, causing more pain.” (P05) “Children experience a lot of pain during this process, which makes them dull and withdrawn.” (P05) “Some children resist and refuse to cooperate. We have to approach them patiently, speak to them, and console them.” (P06) “Pain starts right from the beginning for some children, and for a few it becomes very severe, especially with high-dose chemotherapy.” (P08) “Oral mucositis becomes so painful that children cannot eat or drink water. Pain relief becomes necessary.” (P08) “We do not see any pain from outside. But inside the patient has a lot of pain.” (P17) “When the palliative team assesses and starts narcotic medicine, even a child who used to cry all the time starts playing well.” (P17) “If there is mucositis after chemotherapy, the child neither sleeps, nor eats, nor drinks anything.” (P22) | “The biggest challenge we face in the pediatric ward is inserting cannulas. It is a very difficult and painful process for the child.” (P10) “If the child is a baby and the needle is not available, it becomes very difficult to give the injection. The child keeps shaking a lot.” (P11) “Sometimes we insert a cannula one day and it has to be replaced the next day, which means pricking them again.” (P13) “Older children can tell us where they are having pain, but small children will not say anything even if they are suffering.” (P18) “Children who undergo chemo get irritated. Mothers become tense because of the child’s behaviour, but it is because of pain and weakness.” (P19) “Earlier there was an issue with IV lines, so many times the child had to be pricked. That did not feel good.” (P21) “After palliative medicine is started, children get relief. They can sleep and there is no pain.” (P22) “When we go to give an injection to a small child, he cries a lot and does not let it be given.” (P23) “Older children understand more and their questions are tough. Managing their pain and emotions is challenging.” (P24) “Ninety percent of the time, pain is the main problem. Once pain is controlled, other issues reduce.” (P15) |
| 1.3 Treatment continuity across care settings | “Besides chemotherapy, sometimes we perform blood transfusions, intrathecal injections, or bone marrow procedures. After intrathecal injections, we observe the child for an hour.” (P01) “Mainly chemotherapy procedures, managing fever, administering antibiotics, setting up IV lines, and for some children, providing chemo through a port.” (P02) “When we start chemotherapy, we check the IV line to ensure there is a proper backflow… If there’s no backflow, we replace the line before proceeding.” (P05) “It’s not just about administering the medication; it’s also about constant observation, communication with parents, and addressing any adverse reactions immediately.” (P05) “For fever or BP-related emergencies, we administer injections as needed and monitor the child’s condition every half hour.” (P05) “I usually start my day at 7:30 AM… I take their vitals, ask them if they’ve had food, if they’ve passed motion, or if they’re in pain… and report to the doctor.” (P06) “We administer chemotherapy, blood transfusions, IV antibiotics, and sometimes immunotherapy or IVIG depending on the patient’s needs.” (P06) “Blood transfusion means… we first check the vitals… if the child has fever or shivering… we stop the blood and inform the doctor.” (P18) “After that, we start for injections… IV fluids, chemotherapy, then IT, then the doctor’s rounds… discharge procedure… and then handover.” (P23) “Then we start the chemotherapy. We secure the IV line first… We need to be careful about extravasation.” (P01) | “Regarding additional treatments… we mostly operate as a daycare unit… If anything serious arises, like glucose drips, we advise on that.” (P03) “For mouth sores, we recommend using chlorhexidine mouthwash regularly…” (P03) “If they’re unable to drink a lot of water, we encourage small amounts slowly and frequently.” (P03) “If new medicines are added during rounds, we administer them promptly unless there’s a delay in stock availability.” (P05) “A typical day starts with checking all the rooms… bed-making, medications, vitals…” (P10) “Now I work in OPD… patients get chemo in day care or have investigations and go home.” (P17) “I start with medication… chemo chart… IT process… blood transfusion, platelet transfusion…” (P21) “After administering the injection, we observe them for 10–15 minutes before letting them go.” (P01) |
| 1.4 Comfort, dignity, and daily function during intensive treatment | “Even when children are on a drip, they take the cycle and move around, play, and spend some time comfortably with us.” (P04) “We inquire if they have any issues, if they have eaten, how they are feeling, and if they have slept well.” (P07) “Nutrition is a major focus, and pain management is another important aspect, as chemotherapy often causes issues like mucositis.” (P06) “If they have mucositis, we provide education about mouthwash, personal hygiene, and gargling.” (P07) “Mostly, we advise children to drink a lot of water and keep their mouths clean. If the problem doesn’t subside, we admit them and give supportive medicines.” (P03) “My round starts by asking if the child has eaten and if the mother has slept. If not, we let the mother rest for some time.” (P20) “Sometimes children want only specific food, so we try different ways to make something they will eat.” (P22) “We explain to the child and parents, talk to them, reassure them, and then inform the doctors if changes are needed.” (P24) “However, once a child becomes familiar with our staff, they recognize us and become more comfortable.” (P01) “Sometimes children get scared seeing our uniforms, so we talk to them lovingly or give them a chocolate.” (P18) | “We must handle their care carefully to avoid any issues and ensure they are comfortable.” (P01) “Initially, they are very nervous. They cry a lot and scream, but when we talk to them supportively, they slowly get convinced.” (P02) “By the next treatment cycle, they are no longer afraid of the treatment or the injections. They come for chemotherapy willingly.” (P02) “Almost all children cry when they first come, and they’re scared that the nurse will give them an injection.” (P02) “They remember us and ask about our well-being, like if we’ve eaten or not. After becoming close with them, their fear decreases.” (P02) “When children who have been here before return, they recognize us and realize that we are only trying to help them, even though they might still cry or resist a little at first.” (P03) “We have playrooms here, where we try to divert their attention and make them feel comfortable. We do our best to support them through this journey.” (P10) “We instruct them to drink plenty of water before and after the chemotherapy to avoid dehydration and reduce the risk of complications.” (P02) “We also provide education on mouth hygiene, mouth rinses, and managing pain.” (P02) “When they come with vomiting or other issues, we administer medicines, such as antiemetics.” (P03) “If they are extremely weak, we insert a cannula and provide IV fluids.” (P03) “If they have issues during bowel movements, we give them betadine solution.” (P03) “If the baby has loose motions, then the dietician may suggest that the baby should be given rice water or dal water.” (P20) “That is my job to make it available according to the requirement of the diet.” (P20) “Children who come from home are used to top feed, but after coming here, they have a little problem to manage.” (P17) “Their feeding timing changes, and it becomes difficult for very young children.” (P17) “One patient was admitted for three months, and he wanted to eat cake only.” (P22) “The cake was not available, so I searched and made a wheat cake.” (P22) “After I fed him, only then he calmed down.” (P22) “We provide additional services to the family.” (P23) “We give ration to families who come from other places.” (P23) “We also provide money and food, because parents have to eat and the child also has to eat.” (P23) “Even though we have workload, if two kind words make the child feel better, then we should say it.” (P24) “We should do it for them.” (P24) “At least they should get some relief according to their condition.” (P24) “Whatever is related to the disease happens, we try to reduce the problem.” (P24) “After palliative care started, the children got relief.” (P24) “They remained calm and could sleep.” (P24) “Their parents also felt relaxed.” (P24) “Sometimes he also needs a wheelchair after an operation, that should also be given to him.” (P24) “Sometimes he is handicapped, sometimes a handicapped child may be born after an operation, that too should be given to them.” (P24) |
| 1.5 System and safety constraints in nursing work | “Then we start the chemotherapy. We secure the IV line first. We ensure the IV line is comfortable and not placed in a thin vein. We secure the IV line and administer chemotherapy. We need to be careful about extravasation.” (P01) “When we begin chemotherapy, we wear protective gear such as goggles, caps, masks, sterile gloves, and gowns for biosafety.” (P05) “Before administering any drug, we conduct a test dose and wait for about half an hour to monitor for any immediate reactions.” (P05) “Because of that, exposure causes leg pain, body pains, headache, hairfall, there are many complications.” (P12) “Cleanliness first, they have very low immunity powers, it is better if the entire area is clean.” (P12) “One of the major issues here is related to hospital management, particularly in terms of sanitation and hygiene.” (P10) “Needs means they should get proper hygienic treatment, but even if we tell them, the patients never listen.” (P21) “If we are not properly hygienic, then the treatment will be meaningless.” (P21) “Our ward is quite small, with limited resources. We have only 10 beds.” (P05) “Additionally, we are short-staffed. Having more staff would make it easier to manage.” (P05) | “Protocol means chemo protocol. After the chart comes, one should go at the same time.” (P21) “Precautions should be taken by wearing cap, gown, mask, meaning the patient should not get any infection.” (P21) “There is often inadequate water supply, especially in areas where children are dealing with infections or frequent motions.” (P10) “There are issues with washrooms or bathrooms that might not always meet their needs.” (P14) “In such cases, we guide patient families to stay in nearby inns or accommodations where the environment is more hygienic.” (P14) “On top of that there are some parents that if we tell them not to crowd here, they point fingers at each other.” (P23) “Yes, father needs a place to stay. It is cold, there are mosquitoes, and some people have even got dengue.” (P23) “Sometimes, when the stock runs out, attendants have to buy medicines from outside.” (P14) “It would be helpful if all necessary medicines were always available in the hospital.” (P14) “If our ward is full, our patients go to other wards and they face problems in everything from food to drink.” (P17) “There’s also a lack of security, especially at night, and parents don’t feel secure.” (P14) “It is better if there is one counselor for a ward.” (P12) “Sometimes we cannot use advanced treatment methods like ports, so we have to rely on IV lines, which can be painful.” (P08) “Some facilities are advanced, but not everything is available.” (P08) “Sometimes he also needs a wheelchair after an operation, that should also be given to him.” (P24) |
| 2.1 Shared understanding of illness, treatment, and prognosis | “Parents, when they come initially, tend to feel frustrated and overwhelmed. Over time, we show them other children who have completed treatment and explain how they’ve recovered. We also explain the side effects of chemotherapy so they are prepared. Gradually, they start sharing their problems and develop a close bond with us.” (P03) “During treatment, parents ask why their child is receiving a certain treatment and why it differs between children. We explain that every case is different, even if the medication is the same, and try to explain it in a way they understand.” (P03) “Sometimes parents are educated and understand instructions quickly, but many families come from rural areas and lack awareness. For them, we need to explain repeatedly and clearly.” (P05) “In the beginning, they don’t know anything about the ward or the treatment. But as we explain things step by step, they begin to grasp the situation better.” (P05) “The first challenge is diagnosing the illness. Many times, symptoms appear as just a headache or fever, and parents think it’s a normal fever.” (P08) “Daily counseling is essential. Every day, there’s someone with a different issue.” (P09) “A mother might say, ‘I don’t want to continue chemo because my child’s counts are low.’ She doesn’t understand that chemotherapy reduces both good and bad cells.” (P09) “They compare themselves to others in the ward and start believing they’ll have the same outcome as the patient next to them.” (P09 “Most caregivers don’t understand who does what. They think the nurse gave treatment and the doctor prescribed something, but they don’t realize palliative care is also involved.” (P09 “Older children think being sent to palliative care means treatment is over.” (P19) “Usually there is not much resistance. Parents may ask why palliative care is needed, but once we explain the benefits, they generally agree.” (P10) “If parents are told, ‘Your child might not recover,’ it gives them a chance to prepare emotionally.” (P14) “After relapse, parents panic and say, ‘I did everything for my child, still the disease came back.’ They ask us, ‘Sister, what should we do?’” (P22) “Some families become emotional and relieved when palliative care is explained, but others don’t understand it at all, and it becomes very challenging to explain everything clearly.” (P05) | “I don’t have much experience explaining it, but I think parents might not fully understand, as it’s a different kind of treatment.” (P03) “Sometimes it can be challenging. Even though we explain, some parents don’t understand right away. They expect quick results, and when things take time, they become anxious or frustrated.” (P03) “We explain this to the children in a way they can understand. If they don’t drink enough water, it affects urination, appetite, and can cause burning or sores in the throat.” (P04) “It’s not like what’s written in the book. If you just tell them book language, they won’t understand. When we explain using what we see daily, they accept it better.” (P04) “When we see other children, oncology patients feel more discomfort. Families worry when their children lose hair and often don’t understand the procedures fully.” (P08) “Parents often think the child should only drink milk or liquids during fever, and they fail to provide proper nutrition.” (P09) “Some parents listen and understand, but others remain skeptical even after repeated explanations. They believe it only when a doctor or counselor says the same thing.” (P09) “Small children think they have come only to get an injection and nothing more.” (P11) “Parents don’t know what ‘Positive’ means. That’s why we say there is a Madam specially for pain.” (P11) “They take the treatment, but they don’t know how to take medicines properly or at the correct time.” (P12) “Even after five cycles, their understanding in the first cycle and later cycles is often the same.” (P12) “They sign consent because it’s part of treatment, but they don’t know what side effects will come after the first chemotherapy.” (P17) “They didn’t understand the first counseling as much as they understood later.” (P17) “Parents often explain to other parents that this happened to my child, so it will happen to your child also.” (P18) “We take both parents aside and explain that every child is different.” (P18) “What all answers will we give to mummy now?” (P19) “We have to be a mummy too.” (P19) “She was just having fever. She never had this before.” (P19) “Sister, children do get well, right?” (P19) “Can such a big cancer happen just because of a fever?” (P19) “Parents become anxious when they see vomiting, rashes, and hair loss.” (P19) “They mostly don’t know anything about Bombay, and because of that, they have language problems.” (P20) “If they don’t understand the language, we first make them sit and calm down before explaining.” (P20) “Parents need daily psychological support.” (P15) “Some parents understand, some don’t.” (P23) “Young children don’t understand treatment, but older children cooperate when they know their pain will be managed.” (P01) “Even after going home, parents don’t understand what happens inside the body. Everything depends on reports like CBC, which they don’t understand.” (P22) “Both parents were educated but not ready to accept the diagnosis. Seeing other children made the mother insecure, but slowly she began to understand.” (P22) “Many people resist palliative care at first, but with proper education, they may accept it.” (P07) “After treatment failure, parents hold onto hope and want to continue treatment rather than accept palliative care.” (P07) “Parents deny prognosis and say, ‘We believe in God. Our child will get well.’” (P23) “The mother said, ‘Don’t explain this to the child,’ even when the palliative team advised how to talk to her.” (P23) “Patients should be clearly told how much medicine to take, when to take it, and not to exceed the dose.” (P12) “If you send patients without explaining, they won’t understand why they are being referred.” (P12) “Duty doctors don’t come regularly, so attendants question medicines and feel unsure about what is being done.” (P14) “We are busy and don’t always have time to explain everything, but palliative care staff know how to explain clearly and reassure families.” (P05) “Bonding is important because patients come with very little information, but we don’t have enough time to counsel everyone properly.” (P09) “One counselor and one nurse are not enough to meet families’ needs.” (P09) “From the start, families should be counseled about disease, treatment, prognosis, and financial support before admission.” (P12) “Earlier, daily counseling improved care quality. Now that system is reduced.” (P14) “Bed-to-bed counseling is almost nonexistent now, and families don’t get enough time to understand.” (P14) “New families are confused and overwhelmed and often misunderstand what they are told.” (P14) “Counseling happens only at the beginning. When parents hear about cancer, they become lost and frustrated.” (P17) “At the start of treatment, patients really need someone to guide them, or they may drop out.” (P17) “After cancer diagnosis, some families face stigma. Neighbors don’t allow their children to play together.” (P19) “An adolescent told us not to inform his father about his condition because he didn’t want to upset him.” (P23) “Parents lose sleep, face money problems, and start fighting. Sometimes both need counseling.” (P23) “Parents compare their child with others and accuse staff of giving wrong injections or higher doses.” (P23) “It is very challenging to tell a mother that her child is dying. We cannot say it directly.” (P23) “The palliative team explained the situation directly, and the mother said she understood, but we cannot communicate like that ourselves.” (P23) “After the child died, the mother blamed the palliative team for telling the child the truth.” (P23) |
| 2.2 Trust, familiarity, and continuity in care relationships | “Once a child becomes familiar with our staff, they recognize us and become more comfortable.” (P01) “Initially they are very nervous and cry a lot, but when we talk to them supportively, by the next cycle they come for chemotherapy willingly.” (P02) “When children who have been here before return, they recognize us and know we are trying to help them.” (P03) “We see them repeatedly, and over time they and their families start to feel like part of our own family.” (P05) “When they see familiar faces, they cooperate better. Sometimes we are the only staff available, so rapport becomes essential.” (P01) “They remember us and ask about us, ‘Did you eat?’ After becoming close, their fear reduces.” (P02) “Children forgive quickly. Even if they cry during a procedure, they come back to talk or play with us.” (P03) “They ask where we were if we take leave. If we don’t come one day, they notice immediately.” (P20) “Parents feel they can share their inner feelings with us because we are in front of them every day.” (P18) “When admissions last for months, we talk like a family. We tell mothers at night, ‘You sleep, I will look after your child.’” (P24) “Some children come back after discharge and still talk to us. It feels good to see how they have changed.” (P02) “Even years later, old patients come and meet us. Their parents still remember us.” (P24) “We talk to adolescents like friends. They tell us about college, friends, even personal things.” (P18) “Children share more with nurses than with doctors because we remain closer to them.” (P22) | “It takes one or two days for them to adjust. After that, they start interacting nicely.” (P08) “Younger children get very attached to us, but children between 5 and 12 years are less cooperative.” (P04) “Some children hug us when they come. They call us ‘Didi’. I cannot forget those moments.” (P21) “When children are dull, we try to engage them like our own kids, making them talk or smile.” (P06) “Parents feel confident when someone is there for them, especially during night duty.” (P24) “Recovered children send photos and updates. Mothers still call us.” (P19) “Even after growing up, they recognize us and come to meet us.” (P24) “Some children open up slowly. At first they are silent, but over time they start talking.” (P06) “We share jokes, do good-morning wishes. It becomes family bonding.” (P21) “If we are calm and friendly, children who are scared of the uniform also relax.” (P20) |
| 2.3 Counseling and care navigation for treatment continuity | “They think, ‘We came for one chemo and now she seems fine.’ They don’t understand the disease isn’t cured with one chemo, so they stop coming and the disease comes back.” (P09) “Some children became normal and went to maintenance, but after going home they were not taking medicines properly and then suddenly they relapsed.” (P12) “Starting people should be told everything, disease, treatment, prognosis, funds, before admission. Admission should happen only after counseling.” (P12) “When it is the first start, the patient really needs someone to guide them. Otherwise, the patient leaves without proper guidance.” (P17) “New patients are very confused and overwhelmed. Even if we tell one thing, they may understand something else.” (P14) “Daily counseling is essential. Every day there is someone with a different issue.” (P09) “Earlier, counselors used to call families before reviews. That follow-up system made a huge difference. Now it is missing.” (P14) “There is a big gap between treatments because families delay visits. That delay causes complications and pushes treatment out of control.” (P09) “When parents talk to other parents who have progressed one cycle ahead, they are less likely to drop out of treatment.” (P17) “We have sessions where counselor, dietician, doctor, and nurses explain everything together. Old patients demonstrate things, so new families learn better.” (P20) “We explain daily hygiene, medicines, when to call the doctor, and not to ignore loose motions or fever.” (P17) “Parents feel comfortable sharing their inner feelings with us because we are in front of them every day.” (P18) | “It is not like blood cancers are not reversible; there are chances for relapse. Some children take half treatment elsewhere thinking it is enough.” (P04) “Most children complete treatment, but some go back to their hometown and don’t return for follow-ups.” (P04) “There is a need for awareness about nutrition, timely reviews, and follow-ups. Even after explaining, their mindset doesn’t change.” (P09) “I might call someone for treatment, but they don’t come on time. Later they say they weren’t given medicine.” (P09) “I have seen many patients recover because they follow treatment properly. Those who delay face complications.” (P09) “This is a day-care unit with a time limit. Some patients come late and then reactions happen.” (P17) “Many families don’t cooperate in the beginning. By the second or third admission, they start understanding better.” (P05) “Parents from rural areas don’t understand what is going on. They have to be taught again and again.” (P22) “We provide health education on what to do after chemotherapy and how to manage at home.” (P01) “We explain side effects, diet, hygiene, and give specific advice to parents.” (P01) “Initially, parents are under immense stress and not in the right state of mind to understand.” (P10) “Follow-up is very important, but because patient numbers are high, someone always gets left out.” (P09) “Before starting chemotherapy, we hydrate the child and explain why this is needed.” (P05) “If parents don’t understand, we take both aside and explain properly.” (P18) “Some parents want to go home after half chemo. We explain admission is needed.” (P18) “Parents are scared of ICU. We explain it means better monitoring.” (P18) “Palliative team explains very well, diet, emotions, and our workload reduces.” (P18) “Even if the condition is very bad, counselling helps families continue care.” (P18) “Many parents don’t know what palliative care is. After meeting once, nobody refuses again.” (P18) “Parents are worried when chemo is stopped or antibiotics are started. We explain why.” (P22) “Some parents search everything on Google. After explaining calmly, they accept.” (P22) “Sometimes I write medicine timing on paper and ask them to call the on-call number.” (P22) “Only after repeated counselling and a home visit did they start giving morphine properly.” (P22) “Older children sometimes teach their parents how to take medicines.” (P23) “We give examples of children who recovered and grew up.” (P23) “Some parents compare children and accuse staff. We explain every child is different.” (P23) “Parents manage children at home and in hospital together. That creates stress.” (P24) “At night, we tell mothers to sleep and assure them we will look after the child.” (P24) “If we explain palliative care clearly, families accept it.” (P04) “We explain using what we see daily, not just from books.” (P04) “If families don’t understand, we call the counsellor ourselves.” (P14) “Counselling should be done jointly at diagnosis.” (P20) “Parents trust survivor families more.” (P20) “Palliative care does not mean treatment is over.” (P19) “After meeting palliative team, mothers become calm.” (P19) “Pain can come anytime. That is why palliative care is needed.” (P09) “Families accept palliative care better when the team is familiar.” (P10) “If families need time, we continue routine treatment and counsel again.” (P24) “Palliative team should be available at night.” (P11) “Palliative care nurses manage pain and support emotionally.” (P09) “When pain is not controlled, palliative infusion gives relief.” (P01) “Good teamwork keeps treatment going.” (P10) |
| 2.4 Preferences, expectations, and perceived quality of care | “Parents have a very positive opinion of palliative care, especially regarding pain management. When they come next time, they ask for the same medicine. Parents don’t want their children to experience pain.” (P02) “When pain is not controlled, palliative care infusion gives major relief.” (P01) “If we explain palliative care clearly, families accept it. There is no resistance to pain medicines.” (P04) “People who don’t have money can get free treatment here. Arogyashree and trusts help with money, food, clothes everything.” (P11) “Here the whole treatment is free, but patients do not come for maintenance. They think if it is free, nothing will happen.” (P12) “We talk to them about school, friends, and give them books or storybooks so their mind gets diverted.” (P04) “Even with IV lines, children play, move around, and sit with us comfortably.” (P04) “After a few days, children start saying what pain they have and what medicine they are getting.” (P11) “At night, we tell mothers to sleep and assure them we will look after the child.” (P24) “Reducing pain and constipation improves how families feel about care.” (P15) | “Parents don’t want their children to experience pain.” (P02) “Pain relief makes parents feel reassured.” (P02) “All chemotherapy, scans, and investigations are available here. There is no need to go outside.” (P15) “Poor and uneducated patients don’t know what support is available. That must be explained clearly.” (P21) “We use tricks or distractions to make the process smoother for children.” (P03) “Painting and creative activities help distract children and lift their mood.” (P06) “Children need entertainment, books, drawing, mobiles. That helps them feel better.” (P23) “Palliative care people give books or drawing materials and talk nicely.” (P23) “It takes one or two days for children to adjust. After that, they start interacting nicely.” (P08) “Children understand their medicines and schedules better than expected.” (P11) “Some children tell us directly what injection they need.” (P23) “Older children search on Google, so we explain using examples of recovered children.” (P23) “Parents have to manage most of it and make the child understand.” (P23) “Some parents understand and think positively, some don’t accept even after explanation.” (P18) “We teach families intake–output, hygiene, diet, and safety.” (P05) “We explain side effects so parents can observe and report early.” (P05) “With training, nurses can support families better.” (P13) “Many people don’t know pediatric palliative care exists.” (P13) |
| 3.1 Everyday palliative support within nursing care | “In end-of-life situations, we provide emotional support and try to comfort the families, giving them courage.” (P02) “We try to console them, explaining that nothing else could have been done and that their child is no longer suffering.” (P03) “Sometimes, a mother might come alone with her child, and suddenly, something happens.” (P05) “Even for us, managing such situations in our busy schedules can be very hard.” (P05) “In pediatrics, during the end stage, we need to be attentive to their requests.” (P07) “Palliative care means providing psychological support to patients and ensuring they experience minimal pain.” (P16) “Apart from pain management, the patient should be happy till the last moment.” (P17) “They go to the patient’s home and support them even there.” (P19) “After death, till the body is handed over, I go with them.” (P20) “Then the palliative team later gave a home visit to their house.” (P22) | “It feels very emotional because these families have been with us for a long time.” (P03) “We talk to them in a way that gives them some peace and comfort.” (P03) “The most challenging phase is handling death situations.” (P05) “These mothers often don’t have anyone to rely on back home.” (P05) “We should provide support through our team, ensuring they feel happy and understood.” (P07) “I didn’t receive any palliative care training, but after coming to MNJ, I’ve been aware of palliative care for the last 17 or 18 years.” (P15) “I’ve seen patients who are in peaceful death, and those who aren’t.” (P15) “Why would I stop caring for my patients at the end?” (P15) “Would I leave them to suffer?” (P15) “Pain and palliative care are definitely important for those patients.” (P15) “We offer good treatment to children, talk to them, and help them understand their condition.” (P16) “Everyone has a desire, even a little girl.” (P16) “In end-of-life care, the palliative team sometimes goes to homes to provide moral support and psychological support.” (P17) “When the palliative patient does not come to OPD, the palliative department visits the patient at home and gives medicines according to the condition.” (P17) “If the patient is in pain, then they can reduce the pain by getting involved themselves.” (P18) “At the end moment, they can support the mother and father by counselling them well.” (P18) “They take care of them even after going home.” (P19) “If someone needs oxygen or an ambulance, they try to make that available.” (P19) “I give some peace of mind to the parents.” (P20) “Losing a child is very difficult because one is so attached.” (P20) “I sit quietly.” (P20) “Only then they were taking the medicine.” (P22) “Only then the girl was fine.” (P22) “After treatment failed, he died but his mother and father still call us.” (P24) “They have just given birth to a second baby.” (P24) “Till then his routine treatment is not stopped, it continues.” (P24) “The doctor gives families time to decide on palliative care.” (P24) |
| 3.2 Late recognition of palliative needs | “Many people come to us only after their loved ones are in the final stages. If they had been informed earlier, they might have had better control over the situation.” (P13) “Sometimes the patient has fever, sometimes convulsions… parents get worried… so we have to counsel the parents.” (P18) “They think, ‘Why do I need this? I don’t even have pain.’ What they don’t understand is that pain can come at any time.” (P09) “Even though there is a perception that palliative care is only for end-of-life situations, it is not always the case.” (P01) “They don’t realize that palliative care is also involved and provides crucial support for the patient.” (P09) “If there’s a treatment failure, they may not accept it… they want to continue treatment rather than switch to palliative care.” (P07) “Older children think being sent to palliative care means treatment is over.” (P19) “We don’t say that we have referred you for palliative treatment… the madam will explain it to you very well.” (P18) “Why do I need palliative care, it was fine right now. His reports were fine right now.” (P24) “Let the senior doctors come first… after consultation with all the doctors, they become ready.” (P24) | “Some parents say that they didn’t fully understand what palliative care is. They feel that it’s not as effective as other treatments, and it makes them feel a bit disappointed.” (P03) “I don’t have much experience explaining it, but I think they might not fully understand, as it’s a different kind of treatment.” (P03) “Mostly I have seen palliative, but many people have asked that Baba what is this Palliative.” (P18) “Palliative means I heard it only after coming here, I didn't know that there is palliative care first of all.” (P12) “Sometimes the patient suddenly gets fever, sometimes he gets a bruise. So, what has happened? Sometimes parents say what has happened?” (P18) “Parents get worried that why did this happen to him? So we have to counsel the parents.” (P18) “Some people have this fear in their mind that we took treatment here, why do we need to go there for palliative.” (P22) “So people get scared, what is the point of it now… they mean it is a later part of the treatment.” (P24) “If madam tells them that they have to go for palliation, or we explain to them, they would not accept it.” (P20) “When a half patient becomes normal and sometimes comes in such a relapse, then they are afraid.” (P20) “Because they are never willing for further treatment.” (P20) “Older children then ask if it means there is no treatment for it.” (P19) “They search on the mobile that there is no treatment.” (P19) “After meeting you once, no one said that I will not go back.” (P18) “After the second or third day, okay as you do doctor, okay.” (P24) “For them, it’s just, the nurse came and gave treatment, the doctor prescribed something.” (P09) “They just felt better because of the medicine that the doctor prescribed, and because of it, my pain was reduced.” (P17) “Palliative care is mainly for those at the end stage.” (P07) “Sometimes at the last moment the patient understands what palliative means.” (P17) |
| 3.3 Care reconfiguration following palliative involvement | “There are many NGOs in our department who help families, and because of them even uneducated patients complete the 2–2½-year treatment well and go home.” (P17) “Parents work very hard for their child but don’t have proper washrooms or a place to rest. Even in such big wards, there is no comfortable space for them to lie down or freshen up.” (P17) “Mother and father sit by the child 24/7, often sleeping in chairs all night without proper rest, especially those coming from long distances.” (P17) “If they need platelets or blood, parents get very tired and irritated because they already have low income and have to keep paying again for repeated investigations and transfusions.” (P17) “The palliative team even goes to patients’ homes, arranging things like oxygen or an ambulance if needed, and continues supporting them as long as the patient is alive.” (P19) “Social workers help families get cheaper medicines through ClearPoint or Tata funds, guiding them about documents like ration card and Aadhaar so they don’t waste money and time buying at full cost.” (P20) “For outstation families, social workers arrange free, safe rooms and a vehicle so parents can stay nearby and come any time there is a problem.” (P20) “A pediatric day-care unit was opened two years ago so children can get outpatient chemotherapy and reduce admission costs.” (P01) “In my opinion, almost everything is provided here nutrition, pain management, and even financial help through social workers which is very helpful for families.” (P06) “Poor patients from villages get free treatment through Arogyashree and different trusts that provide money, food, and clothes, so they can continue cancer care.” (P11) | “What is the helpline in Tata Hospital? Similarly, we need a helpline here because our first-time patients are admitted for 10–15 days and need guidance.” (P17) “Food comes twice a day and that can be adjusted, but hygiene issues and the lack of a proper resting place mean parents don’t get the rest they need.” (P17) “They get breakfast and lunch from outside NGOs like Lions Club and Rotary Club, so at least they don’t have problems with their own food.” (P17) “Space should be given first for relatives who come from far away; those who live nearby can go home, otherwise the rest area and washrooms become too crowded.” (P17) “We explain hospital rules and regulations to families and guide them to places where they can bathe and maintain hygiene, with help from social workers.” (P17) “Our children’s shelters are linked with an organization that gives letters for accommodation, so families can bring their belongings and stay there.” (P18) “Sometimes they have no funding to buy medicines, so madam arranges some money and social workers guide them; it gives parents confidence that someone is helping them.” (P20) “We have most services—nutrition-related support, a palliative care team, and even funds—already available for families.” (P21) “The treatments here are good and we have no complaints about medical care, but hygiene and sanitary conditions are still a priority need.” (P10) “Those people from the trust come to our hospital and help poor patients who don’t have money by arranging blood and other services for free through the social worker.” (P11) “Treatment is going well and food and shelter are good, but buying blood and platelets from outside is very costly—SDP can cost 10,000 and if you need it twice weekly it can reach 20,000, so donors should be encouraged more.” (P12) “Some attendants don’t know where to find services like blood banks in Hyderabad, so we direct them to nearby or public blood banks because 1 SDP can cost up to ₹15,000, which many families cannot afford.” (P13) “The inn is like a hostel with no fee, where they provide accommodation, fresh food, and free services; it’s not luxurious but it’s a comfortable and practical option for poor families.” (P13) “All chemotherapy and scans are available free in the government sector here, and even palliative and radiation-related treatments are handled within the hospital, so there is no real need to go outside for treatment or food.” (P15) “Others tell them to go to the blood bank, and we communicate through the founders who help in blood-related matters for patients.” (P16) “In this new building there are water problems, so providing more support for water would really help families.” (P16) “Palliative care is accessible because they come on daily rounds, so support is always available when needed.” (P16) “They get breakfast and lunch from outside organisations, so they do not have any problem with their own food.” (P17) |
| 4.1 Emotional labour in everyday clinical care | “The child is in front of you, you cannot cry in front of him, because he will be more scared.” (P20) “It feels difficult seeing children in such conditions. I often think about how it would be if my own children faced these problems. We also form a bond with the children. We need to listen to the pain of their families too.” (P02) “Yes, sometimes we get emotionally attached to the children. They've been with us for a long time, and it's hard to see them suffer.” (P02) “Seeing children of the same age as our own children is genuinely difficult. Detaching emotionally is not possible.” (P04) “It’s very hard when something bad happens. We feel deeply sad, but we can’t show it to the parents. We explain everything with care and tell them to stay strong.” (P13) “Even if we feel emotional inside, we still have to continue our duty.” (P16) | “That we go with positive, keep some prayer. The doctor who is explaining in front of you, you have to take positive from this, otherwise no, he said this, he said that, we don’t have to go into rumours.” (P20) “If we talk loudly, they think that this sister is not good enough. We have to deal with it calmly first.” (P20) “When a child is shifted to ICU, then there is fear in those cases.” (P20) “Some parents are very disturbed and keep asking questions again and again, especially after relapse.” (P24) “Sometimes families do not support them. Neighbours don’t allow children to play.” (P19) “We tell them this disease can happen to anyone.” (P19) “Sometimes we relate these children to our own life situations.” (P18) “The closer we get to them, the closer they become with us.” (P02) “The kids call us by names like ‘big sister,’ ‘aunty,’ or ‘mother.’” (P04) “With chemotherapy, we have to insert lines repeatedly.” (P05) “Seeing children in pain or discomfort is emotionally tough at times.” (P03) “When we talk to them supportively, they slowly get convinced.” (P02) “Almost all children cry when they first come.” (P02) “Before giving injections to small children, it becomes very difficult.” (P11) “Sometimes children are dull, and we try to engage with them.” (P06) “Children think that needles, injections, doctors, and medicines are their whole world.” (P24) “We avoid speaking in a strict tone.” (P05) |
| 4.2 Cumulative emotional strain, moral distress, and coping | “When a death occurs in our ward… it affects us so much that we lose our appetite and sleep, and everything is affected.” (P07) “We used to suffer a lot in the beginning, sometimes for a week. Now with experience, we move past it within a day or two. It has become a habit.” (P15) “In the beginning, it was very disturbing. Seeing these innocent children suffering was overwhelming… later, as the repetition kept happening, the fear reduced a bit.” (P17) “Initially, I was shocked seeing so many children with cancer. Now I have become accustomed to it, but that first phase was very difficult.” (P12) “Despite giving patients all the care and treatment possible, we still aren’t able to save them. That is heartbreaking.” (P02) “We have to accept the situation. There is no other way. That is how we manage.” (P07) “Initially, I used to cry along with them. Later I understood this is my job and I have to carry on.” (P10) “When someone crashes after long treatment, it feels like we are losing somebody… that day we don’t think positively.” (P20) “Even after going home, I used to think about why this happens only to children.” (P18) “With age and experience, mentally we have grown stronger. Earlier it affected us for many days, now we recover faster.” (P15) | “It feels good when we see children recovering… but some days, it even makes us not want to come to work.” (P08) “Deaths are increasing from 2–3 months… I feel very distressed despite treatment going on.” (P12) “He was with us for one and a half years… when I took him in my lap, I felt like crying a lot.” (P21) “In pediatric cases, the impact is different… we feel disturbed again and again.” (P15) “Seeing children who are the same age as our own… it doesn’t happen that we can detach.” (P04) “Sometimes it comes to mind what we could have done differently, but later we feel this is the way of the world.” (P20) “These are cancer patients. We cannot guarantee tomorrow. We do as much as we can for them.” (P17) “When I go home, my daughter understands from my face that something is different… that helps me accept that we did everything.” (P20) “We carry these memories home. We grow very attached to children who are here for years.” (P13) “Before starting work, I take God’s name… we pray for strength to face this.” (P11) “This is my profession, serving patients is my job, and I’m doing it.” (P16) “After seeing these children, I learned that life should be lived fully.” (P19) “I remind myself that this work is a godsend and we are here for a reason.” (P13) “When the palliative team works along with us, we feel we are not alone.” (P18) “Earlier, we had no counselors… now with counselors and the palliative team, our workload has reduced.” (P18) “If we had more counselors or social workers, it would reduce our burden.” (P09) “When we see children recovering and going back to school, it gives us strength.” (P08) “Parents still call us even after the child has passed away… that connection stays.” (P21) “When parents say thank you, it feels that we have done something meaningful.” (P15) “Seeing children complete treatment and come back healthy makes all this feel worthwhile.” (P02) |
| 4.3 System and staffing pressures shaping emotional labour | “For 100 patients, there are only 5–6 nursing staff. It’s impossible to provide the level of care we should.” (P12) “My interaction with a child may be only 2–3 minutes during cannulation. In that time, we can’t have a full conversation.” (P09) “Sometimes we need to act very quickly, like when a child develops a fever. We must start antibiotics within half an hour.” (P06) “Besides chemotherapy, sometimes we perform blood transfusions, intrathecal injections, or bone marrow procedures… we observe the child for an hour.” (P01) “Once the diagnosis happens, families who are not from Bombay have no arrangement for living or food… we arrange food and guide them for bathing and accommodation.” (P20) “Gents are not allowed inside. They don’t bathe for two to three days… we have to guide them to paid public washrooms.” (P17) “Sometimes we allow extra attendants because of language problems or young mothers… we have to decide who needs more.” (P23) “Once a mother had nothing to eat at night. I shared my tiffin. She said she had no money and was new to Mumbai.” (P22) “Dieticians advise special diets, but there is no free supply. We manage with what we have.” (P08) “Accommodation, hygiene, resting space, these are basic needs. Without this, everything becomes harder.” (P24) | “The children come for chemotherapy on an OPD basis. So, before we arrive, some children have already come, and they wait in the clinic.” (P01) “Each day, children arrive one by one. When they come, the first thing they do is undergo blood tests. We take their samples, label them, and send them for reporting.” (P03) “When we start a shift, the first thing is the handover process. After that, we check vitals for all patients, do file work, give medicines, attend emergencies, and then again handover at the end.” (P05) “We are assigned about 10 beds each, but we take care of all the patients equally. Throughout the day, we attend to patients continuously.” (P06) “If mucositis occurs, we admit them, start IV fluids, give mouth pain management, and if fever arises, we do blood cultures.” (P01) “For fever or BP-related emergencies, we administer injections and monitor the child every half hour.” (P05) “Documentation is very time-consuming. We have to balance documentation with attending to patients.” (P06) “Night duties become particularly challenging because frequent checks disturb the child’s sleep.” (P05) “Our ward is quite small, with limited resources. We have only 10 beds. When there are many admissions, it becomes difficult to manage.” (P05) “We are short-staffed. Having more staff would make it easier to manage and provide better care.” (P05) “We can’t spend enough time with the children or their parents. Sometimes parents want to talk, but an emergency comes up.” (P09) “Our role becomes mainly giving medicines. Psychological support needs more time than we have.” (P09) “If my colleagues are supportive, things go smoothly. If I have to handle everything alone, it becomes very difficult.” (P08) “After dieticians, counselors, and the palliative team came, our workload reduced a little bit.” (P18) “When families come from villages, they worry, where will we stay, how will we survive, Mumbai is expensive. No one knows us here. We have to counsel them about food, rooms, and trusts.” (P22) “Those who are poor face many problems. They cannot afford rooms or food in Mumbai. Even sleeping becomes difficult during admission.” (P24) “Parents coming from Pune, Amravati, Dhule, Nanded face many accommodation problems. We contact shelters through organisations and arrange letters for them.” (P18) “We explain where affordable hostels are. Many families don’t know anything. Explaining reduces their stress.” (P13) “Poor and uneducated patients don’t know what support is available. Even if we explain, they don’t understand easily. That also becomes our responsibility.” (P21) “Children miss home food and stop eating. We coordinate with dieticians, rice water, dal water, whatever is needed, we arrange.” (P20) “Nutrition is essential, but food security is still a problem for some families, even when treatment is free.” (P08) “Parents work day and night for their child but have no resting time anywhere. There should be some convenience for them.” (P17) |
